# Burden of Chronic and Heavy Opioid Use Among Elderly Community Dwellers in the U.S.

**DOI:** 10.1016/j.focus.2023.100175

**Published:** 2023-12-20

**Authors:** Morgan I. Bromley, Easter P. Gain, Mark'Quest Ajoku, Meredith A. Ray, Fawaz Mzayek, Satish K. Kedia, Xinhua Yu

**Affiliations:** Epidemiology, Biostatistics, and Environmental Health Division, School of Public Health, The University of Memphis, Memphis, Tennessee

**Keywords:** Elderly, opioids, chronic, heavy, trends, Medicare

## Abstract

**Introduction:**

Opioid overprescribing may fuel the opioid epidemic and increase the risk of complications of opioid misuse. This study examined trends and determinants of chronic and heavy opioid use among elderly community dwellers in the U.S.

**Methods:**

Medicare Current Beneficiary Surveys data from 2006 to 2019 were used. Common opioid medications were identified in the prescription medication files (*n*=47,264). *Patients with Chronic users* were defined as those receiving 6 or more opioid prescriptions within a year or on medication for 3 or more months, and heavy users were those having an average daily dose of 90 or more morphine milligram equivalents or 3,780 morphine milligram equivalents or more per continuous treatment episode.

**Results:**

One in 6 elderly community dwellers ever used opioids during the study period. Chronic users were more likely to be women than men (68.9% vs 31.1%, *p*<0.001). Of all survey participants, 4.3% were chronic users, and 2.8% were heavy users. Among ever users, 27.7% were chronic users, and 18.1% were heavy users. The rate of opioid use rose from 12.1% in 2006, peaked at 22.8% in 2013, and decreased to 11.7% in 2019. Chronic use was 5.1%, 10.7%, and 7.6%, respectively. Heavy use was 5.5%, 10.7%, and 7.6%, respectively. However, for chronic and heavy users, there was no significant difference in the median opioid dosage and opioid duration between males and females.

**Conclusions:**

Among elderly Medicare beneficiaries, opioid prescriptions have been decreasing since 2013. However, a substantial number of elderly people were chronic and heavy users, calling for better opioid management among them.

## HIGHLIGHTS

• The rate of opioid use among elderly U.S. community dwellers changed between 2006 and 2019.
• Median opioid dosage and opioid duration increased for both male and female chronic and heavy users.
• Those with lower income had higher prevalence, higher dosage, and longer duration than those with higher incomes.
• Those with 2 or more comorbidities had higher prevalence but not higher dosage or longer duration than those with 1 or no comorbidities.

## INTRODUCTION

Opioids are effective medications to manage acute (lasting up to 1 month) and chronic (lasting for 3 or more months) pain, but their overuse has led to a public health crisis. Although opioid prescribing has been declining in the U.S. since 2016, almost 1 in 3 Medicare Part D (drug benefit) beneficiaries filled at least 1 opioid prescription during 2016, and 1 in 10 Part D beneficiaries received opioids for 3 or more months, a more alarming concern.^1^^,^^2^ Studies have warned that longer use of opioids could lead to overdose, misuse, and abuse.^1^, ^2^, ^3^, ^4^, ^5^, ^6^, ^7^ Although opioid misuse and abuse can be seen as the same thing, the difference lies in their intent. Misuse is the taking of medication unintentionally, whereas abuse is the use of medication in an unhealthy or illegal manner.

Yet, opioids, an important pain medicine, are often needed among older people. For example, the median age of diagnosis of common cancers such as lung, breast, colorectal, bladder, and prostate cancers is typically after age 60 years,^8^ and opioids are an indispensable medication for treating cancer-related pain. In addition, the prevalence of chronic pain is 27.6% among people aged ≥65 years.^9^^,^^10^

Opioid abuse and misuse have become a major problem among opioid-naïve patients,^11^, ^12^, ^13^ that is, “patients who have not used opioids for more than 7 consecutive days during the previous 30 days.”^13^ Past research has focused on opioid abuse and misuse mainly among people aged <65 years, whereas among the elderly, previous studies typically relied on Medicare Part D pharmaceutical claims to explore the patterns of opioid prescriptions.^2^ Medicare Part D benefits only cover half of the total Medicare population, and 16% of them are aged <65 years.^9^^,^^10^ There is scarce evidence describing the opioid use patterns among community-dwelling elderly because many Medicare beneficiaries have supplementary insurance to cover drug costs.

It is important to identify and study chronic opioid use and heavy opioid use (defined below) among elderly people because this patient population often seeks long-term opioid prescriptions for acute pain.^14^ However, it is important to remember that much of this population has multiple comorbidities. In addition, many of these elderly individuals may present with poor or reduced cognition, which can impede their ability to properly verbalize or indicate their pain. This can lead to patients becoming chronic opioid users, heavy opioid users, or both. This is compounded by the fact that two thirds of elderly people may have 2 or more chronic conditions and often take multiple medications.^15^ Chronic use of opioids may lead to a higher risk of complications, adverse reactions with other medications, and higher rates of hospitalization or emergency room visits.^15^ Heavy use of opioids may also increase the risk of opioid overdose and induce heart disease and other emergency illnesses in addition to the risks mentioned earlier.^16^^,^^17^ To our knowledge, there is no research explicitly examining the chronic use and heavy use of prescription opioids among elderly people in the U.S.

In this study, we aim to explore the trend of opioid use, duration, and dosage among community-dwelling elderly from 2006 to 2019. We will examine how opioid use changed in the context of the release of the opioid prescription guideline for managing pain by the Centers for Disease Control and Prevention (CDC) in 2016, the enforcement of substance drug prescription management programs in many states, and the issuance of the Comprehensive Addiction and Recovery Act of 2016.^18^^,^^19^

## METHODS

### Study Sample

This study employed a repeated cross-sectional study design. We used Medicare Current Beneficiary Survey (MCBS) cost and use files from 2006 to 2013 and MCBS survey plus cost supplement from 2015 to 2019. MCBS changed contractors in 2014, who also changed sampling and data collection methodologies. Thus, the MCBS 2014 data were not released owing to technical issues making the data not available for inclusion in this report. The data set is public, and all participants are deidentified with a number for their identification. Administered by the Centers for Medicare and Medicaid Services, MCBS is a longitudinal, rotational, and multistage survey to explore the health status and healthcare use of the entire population of Medicare beneficiaries. MCBS collected demographics, medical history, general health status, and healthcare use in each fall interview. The interval interviews predominantly serve to collect healthcare utilization. The MCBS data are also augmented with linked Medicare claims for detailed information about healthcare and prescription medication use during the survey period.

The survey sampling schemes are different before and after 2014, and details can be found on the MCBS website (https://www.cms.gov/files/document/2019-mcbs-methodology-report.pdf). Three interviews, starting with the fall round, were conducted annually over 4 years to track changes in health status and healthcare use among survey participants. In each fall round, patients who have participated for 4 years are rotated out, and new participants of one third of the total sample size are recruited to maintain the representativeness of current Medicare beneficiaries. For each cross-sectional year, two thirds of the study sample are repeated from the previous year.^20^ In addition, MCBS retrofilled a small percentage of participants from follow-up years to ensure the representativeness of total Medicare beneficiaries in the current survey year. The data for MCBS from 2006 to 2013 were based on the ZIP code–based second-stage units, whereas the data for MCBS from 2015 to 2019 were based on the census tract–based second-stage units.

This study cohort consists of community-dwelling elderly Medicare beneficiaries aged ≥65 years. More than 170,000 participants were originally included. However, many participants were counted multiple times because they had multiple visits and prescription records. This number was reduced to 119,964 after lowering each participant to 1 reading and excluding those within the exclusion group. We excluded those who died during the first 6 months since the fall enrollment into the MCBS because their health statuses and healthcare use patterns during the end of life are likely significantly different from those of other participants. In exploring the trend of opioid use among subcohorts such as those who were hospitalized or had a diagnosis related to pain (e.g., cancer-related pain), we further restricted the sample to those in Medicare fee for service for the survey year. Those enrolled in HMOs were excluded because there were no Medicare claims for these participants. In addition, these participants are seen in primary care clinics.

### Measures

MCBS consolidated medication information from self-reported survey responses and prescriptions from Medicare Part D claims into 1 data set. During each interview, participants are instructed to bring or review their medication bottles to verify their current medication use. Drug names, dosages, and days of supply were recorded and mapped to the National Drug Code. All drugs were then reclassified according to drug use standards. Opioids were identified on the basis of the First Databank generic therapeutic class (THRECC group) for pain medicine (group code: 02, 03, 09, 11, 80, 83, 85, 99) and generic drug names (morphine, oxycodone, hydrocodone, tramadol, and other generic drugs). Ever use was classified by whether the participant had opioid use on file or no opioid use on file. Opioid use was further examined as chronic or heavy.

The duration of opioid use was based on the prescription date and days of supply information. Two opioid prescriptions were considered continuous use of opioids if the gap between the end of the previous prescription and the beginning of the current prescription was within 14 days (e.g., from the current prescription date until the end of the last continuous prescription). If the duration of continuous use of opioids lasts >3 months or consists of 6 or more opioid prescriptions within a year, it is considered chronic opioid use (or long-term use in other studies).^4^ The dosage for each opioid medication was converted to morphine milligram equivalence (MME) on the basis of CDC guidelines.^21^ Daily MMEs were calculated on the basis of prescription information. We classified the opioid use episode as heavy use if the average daily dosage of opioids exceeded 90 MMEs or a total of 3,780 MME per continuous treatment episode.

#### Measuring Sociodemographic Characteristics

Each participant's sociodemographic characteristics and general health status were collected during the face-to-face interviews in each fall round during their survey period. We included age at each year (regrouped as 65–74, 75–84, and ≥80 years), sex, income (<$15,000, $15,000–$30,000, and ≥$30,000), race/ethnicity (regrouped as White, African American, and other), marital status (married or living with partner versus other), education (up to some college, associate's, or bachelor's or higher), health insurance status (Medicare only, dual eligibility [Medicare and Medicaid], and other), Medicare Part D benefit (yes/no), and metropolitan status (metropolitan versus nonmetropolitan).

#### Measures

MCBS collected smoking status (current smoker, former smoker, and never smoked) and BMI (weight in kilograms divided by square of height in meters). MCBS also collected self-reported medical conditions such as cancer; heart disease; stroke; arthritis; chronic obstructive pulmonary disease; paralysis/amputation; any bone disease; diabetes; hypertension; any psychiatric disorder; or any neurologic disease, including Alzheimer's disease and dementia. These conditions were inquired as ever had or had past year and were recorded as yes/no in the analytic data. Similar to common comorbidity measures such as Charlson's Index and those based on the National Health Interview Survey, the number of comorbidities was regrouped as 0, 1, and ≥2. Having 2 or more comorbidities was considered having multiple chronic conditions.^22^

### Statistical Analysis

MCBS provided analytical sampling weights to account for the complex sampling design and nonresponse rates of the surveys. Descriptive statistics were based on weighted mean and SE for continuous variables and weighted frequency and percentage for categorical variables. They were compared by student *t* test for continuous variables and Rao–Scott chi-square test for categorical variables between opioid users and nonusers. Cochran–Mantel–Haenszel chi-square test was used for testing linear trends. The time trend of opioid use was modeled using linear regression with an interaction between time and an indicator in the year 2016 to see whether there was a change in the opioid use trend after the Comprehensive Addiction and Recovery Act was passed (interrupted time series analysis). The average opioid dosage and duration were examined similarly. The comparisons and trend tests were further explored for subcohorts for age groups, sex, race, income, metropolitan status, pain diagnosis, and comorbidities.

All analyses were performed in SAS 9.4 using the cross-sectional survey weights. Appropriate subpopulation (domain) analysis was conducted to obtain results for subcohorts. Statistical significance was assessed using a 2-sided *t*-test (or 2-tailed test) with a significance of *p*<0.05. No multiple comparison adjustment was planned because all the outcomes were set a priori. SAS procedures such as Proc Surveyfreq, Surveymeans, and Surveylogistic were used for analysis (SAS Inc., Cary, NC).

## RESULTS

Table 1 presents the descriptive statistics of participant characteristics by opioid use status combining all years from 2006 to 2019. Overall, 15.5% of elderly enrollees used opioids, 4.3% were chronic users, and 2.8% were heavy users of opioids. Of all opioid users, 27.7% were chronic users, and 18.1% were heavy users. Opioid users were more likely to be younger, to be female, to be African American, to have lower income, and to be more likely to have 2 or more comorbidities.

**Table 1:** Characteristics of Medicare Beneficiaries Between 2006 and 2019

Characteristics		Opioid use		Chronic use^a^		Heavy use^b^	
	Total	*n*	%	*n*	%	*n*	%
All	119,964	18,656	15.5	5,146	4.3	3,336	2.8
Sex							
Men	52,967	6,902	13	1,603	3	1,090	2.1
Women	66,997	11,754	17.5	3,543	5.3	2,246	3.4
Race							
Non-Hispanic Caucasian	103,057	15,523	15.1	4,211	4.1	2,787	2.7
Non-Hispanic African American	10,265	2,044	19.9	685	6.7	397	3.9
Other	6,642	1,089	16.4	250	3.8	152	2.3
Age, years							
<75	51,600	7,966	15.4	2,345	4.5	1,669	3.2
75–84	46,836	7,583	16.2	1,931	4.1	1,203	2.6
≥85	21,528	3,107	14.4	870	4	464	2.2
Income							
<$15,000	24,487	5,622	23	2,002	8.2	1,296	5.3
15,000–$30,000	37,017	6,028	16.3	1,733	4.7	1,137	3.1
≥$30,000	58,460	7,006	12	1,411	2.4	903	1.5
Education							
Up to some college	25,754	5,510	21.4	1,883	7.3	1,227	4.8
Associate's	33,256	5,108	15.4	1,409	4.2	909	2.7
Bachelor's or higher	60,954	8,038	13.2	1,854	3	1,200	2
Metropolitan Status							
Not metropolitan	29,595	5,045	17	1,625	5.5	1,079	3.7
Metropolitan area	90,369	13,611	15	3,521	3.9	2,257	2.5
Marital Status							
Not married or divorced	56,379	9,860	17.5	3,079	5.5	1,976	3.5
Married	63,565	8,776	13.8	2,067	3.3	1,360	2.1
Depression							
No depression	109,313	16,069	14.7	4,103	3.8	2,593	2.4
Depression	10,651	2,587	24.3	1,043	9.8	743	7
Arthritis							
No arthritis	54,353	5,333	9.8	938	1.7	938	1.7
Arthritis	65,611	13,323	20.3	4,208	6.4	4,208	6.4
Osteoporosis or broken hip							
No osteo or broken	91,882	12,636	13.8	3,110	3.4	3,110	3.4
Yes osteo or broken	28,082	6,020	21.4	2,036	7.3	2,036	7.3
Number of comorbidities							
1 or no	41,163	3,775	9.2	635	1.5	635	1.5
≥2	78,801	14,881	81.1	4,511	5.7	4,511	5.7

*Note:* All group comparisons have a significance of *p*<0.0001.

a Chronic opioid use (or long-term use in other studies) is considered the continuous use of opioids that lasts >3 months or having 6 or more continuous opioid prescriptions within a year.

b Heavy opioid use is when the average daily dosage of opioids exceeds 90 MMEs or a total of 3,780 MMEs per continuous treatment episode. MME, morphine milligram equivalence.

**Figure 1:**
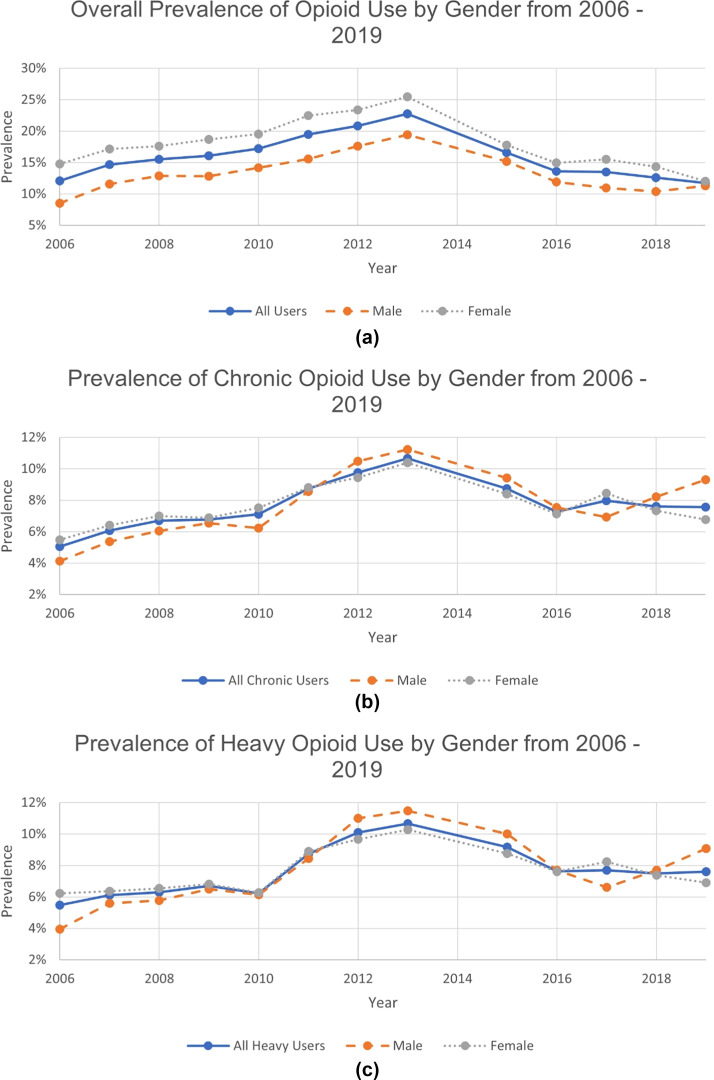
Prevalence of ever use, chronic opioid use, and heavy opioid use by sex from 2006 to 2019. Figure 1a Prevalence of ever use by sex from 2006 to 2019. Figure 1b Prevalence of chronic opioid use by sex from 2006 to 2019. Figure 1c Prevalence of heavy opioid use by sex from 2006 to 2019.

Figure 1A presents the trend of overall opioid use by sex from 2006 to 2019. There was a significant increase in opioid use seen from 2006 to 2013 before decreasing in 2015 when the new guidelines were introduced. The prevalence of ever-opioid use continued to decrease until 2017 when it began to stabilize. The prevalence of overall opioid use was 12.1% in 2006, 22.8% in 2013, and 11.7% in 2019 (*p*<0.0001). This trend was similar between males and females, although females were more likely to use opioids than men. Figure 1B and C shows the trends for chronic opioid use and heavy opioid use by sex from 2006 to 2019, respectively. In both Figure 1B and C, elderly males had a faster increase in chronic and heavy use of opioids than females until 2012 when males surpassed females. By 2017, the prevalence of chronic and heavy opioid use among males became similar to that of females.

Appendix Figure 2A–C (available online) shows the changes in median total opioid dosage during a continuous treatment episode by sex from 2006 to 2019 for overall opioid use, chronic use of opioids, and heavy use of opioids, respectively. Most chronic users had lower median opioid dosage. The trends of overall median opioid dosage were similar between males and females, whereas females had a higher median opioid dosage than males. Male chronic and heavy opioid users had a higher median dosage than female users for several years. Male chronic users had an increase in median total dosage, with a median total dosage of 207 MMEs in 2006 (IQR=138–304) and 280 MMEs in 2019 (IQR=180–360). Females had a median total dosage of 231.5 MMEs in 2006 (IQR=154–330) and 305.5 MMEs in 2019 (IQR=204–360).

Appendix Figure 3A–C (available online) presents the change in median duration (in days) of continuous opioid use by sex from 2006 to 2019 for overall opioid use, chronic use, and heavy use, respectively. In Appendix Figure 3A (available online), a slight increase in the median duration of overall opioid use is seen between 2006 and 2019 for both males and females. Similar to Figure 2B and C, increase/decrease patterns are shown in Figure 3B and C. Female heavy opioid users saw the highest median duration increase with an average duration of 149.5 days in 2006 (IQR=64.5–235.5) and 342 days in 2019 (IQR=136–480). Male heavy opioid users also saw a major increase in median days between 2006 (130 days; IQR=78–186) and 2019 (288 days; IQR=123–245).

Elderly people of younger age (65–74 years) and lower income (<$15,000) had consistently higher prevalence, higher dosage, and longer duration of opioid use than the older age group or higher income (Appendix Figures 4A–C, 5A–C, 6A–C, 7A–C, 8A–C, 9A–C, 10A–C, and 11A–C, available online). There was no significant difference in opioid use among racial groups. Although those with 2 or more comorbidities were more likely to use opioids than those with 1 or no comorbidities, they did not necessarily have higher dosages or longer duration (Appendix Figures 10A–C and 11A–C, available online). Those with 2 or more comorbidities and those with 1 or fewer comorbidities followed the same trend for prescription opioid dosage and duration among chronic and heavy opioid users.

Elderly people living in a metropolitan area had a lower prevalence of opioid use (15% vs 17%) but had dosage and duration of opioid use similar to those of elderly people not living in a metropolitan area. Furthermore, those living in metropolitan areas and those not living in metropolitan areas saw a slight decrease in both dosage (20 for both metropolitan and nonmetropolitan down to 14 and 13, respectively) and duration (15 for both down to 8 and 10, respectively) between 2006 and 2019.

## DISCUSSION

To our knowledge, this is the first study that specifically examined the patterns of opioid use among the community-dwelling elderly. We found that 1 in 6 elderly people used opioids, and there was a significant decrease in opioid use after 2016. However, the trend for opioid dosage and opioid duration appeared to increase slightly even after the CDC/Drug Enforcement Administration drug guideline changes in 2016, especially among chronic users and heavy users.

Consistent with other studies, we found that the trend of overall prescription opioid use increased from 2006 up to 2013 before seeing a slight decline into a plateau.^11^^,^^23^^,^^24^ This stabilization does not bode well for the opioid epidemic, especially among elderly community dwellers. In addition, this plateau was met with the coronavirus disease 2019 (COVID-19) pandemic, which is now known to adversely affect underlying prior conditions. Recent studies have shown an alarming increase in opioid use during the COVID-19 pandemic, but whether this increase has also occurred among the elderly remains unclear.^25^^,^^26^

Many elderly people are considered opioid naïve.^27^ The lack of opioid education leads to several adverse outcomes, including increased pain, constipation, depression, nausea, dizziness, overdose, or death.^16^^,^^17^ In addition, the frequency of doctor visits and cost of treatment markedly increase in this population, which could eventually lead to illicit opioid seeking.^24^^,^^28^ The persistent increasing trends of opioid dosage and duration for chronic users and heavy users described in our study revealed a dire situation that is similar to another published study in which more than two thirds of participants had 5 or more prescription opioids, with the youngest age group (65–74 years) having the highest number of prescription medications at 42.2%.^15^

It is important to study chronic and heavy prescription opioid use in this population for several reasons. First, opioid overdose was more likely to occur among people with long-term use of opioids because the elderly were less sensitive to various signs and may have drug interactions even at relatively moderate doses. These may increase the risk of opioid misuse and abuse or life-threatening opioid overdose.

Second, chronic or heavy use of prescription opioids can lead to further complications in physical and mental health as well as reduce the ability to conduct daily activities and socialize. A major side effect of aging is decreased blood flow and volume, resulting in weakened prescription opioid metabolism.^29^ This metabolic change can lead to insufficient effects of the intended drug and require more opioid prescriptions. Elderly people may also be more likely to have complications (respiratory, cardiovascular disease, and bleeding) even at low or moderate doses.

Third, elderly people with multiple comorbidities often take multiple medications at once, that is, polypharmacy.^30^ Some elderly may be less likely to feel pain or may not be able to properly express their pain (e.g., severity of the pain and the overall type of pain, even after surgeries).^13^^,^^28^ Impaired cognition or dementia hinders the ability to accurately feel the pain or determine how or where the pain originated.^31^ Ultimately, impaired cognition coupled with multiple comorbidities increases the risk of becoming chronic or heavy opioid users.^15^^,^^31^ There is an increased risk of inappropriate opioid use, adverse side effects, interactions with other medications, hospitalization or emergency room visits, and mortality among those who have multiple medications. Thus, it is imperative that healthcare professionals observe and address any changes in opioid use habits among elderly patients to reduce the risk of developing the habits of chronic or heavy opioid use.

### Limitations

Our study has some limitations. The study sample was from a survey. It is possible that individuals with problematic opioid use may be less likely to participate in the survey. In addition, current opioid use was inquired during the face-to-face interview, with no history of prior opioid use mentioned. It was unclear whether some of the interviewees were chronic users. Information from claims may give partial answers to those with Part D coverage, but not all participants had that coverage. There was no information about pain severity in the survey and claims, thus we are not able to judge whether the opioid dose and duration were appropriate. We could not identify whether the participant had opioid misuse and abuse. As stated previously, it is important to remember that much of this population has multiple comorbidities and an increased risk of injury. Both factors play a major role in requiring opioids to treat pain. All the issues mentioned earlier may have led to underestimating the magnitude of opioid use. In addition, it is important to remember that the CDC guidelines observed for this paper are not the current guidelines. The 2022 CDC guidelines were not used for this paper because this paper examined the trend of opioid usage between the years 2006 and 2019. Thus, the trends were examined using guidelines within these years.

There were also several strengths in our study. First, our study cohort consists of nationally representative community-dwelling elderly using MCBS data; thus our findings provided a big picture of opioid use among elderly people in the U.S. Second, we presented the trend and use of prescription opioids by participants’ sex, age, race, SES, and comorbidities and separated by different opioid intake levels (ever use, chronic use, and heavy use). This allows us to identify issues beyond the simple dichotomous indicator of use or no use.

## CONCLUSIONS

In conclusion, our study demonstrated a slight decline in overall opioid use after 2016 among community-dwelling elderly people. However, opioid use remained alarmingly high among elderly people, and the prevalence of chronic use and heavy use increased over time with larger opioid dosages and longer duration. Our results provided concrete evidence to call for policy change in how to optimally manage pain among the elderly.

## CRediT authorship contribution statement

**Morgan I. Bromley:** Conceptualization, Formal analysis, Methodology, Software, Writing – original draft. **Easter P. Gain:** Writing – review & editing. **Mark'Quest Ajoku:** Writing – review & editing. **Meredith A. Ray:** Writing – review & editing. **Fawaz Mzayek:** Writing – review & editing. **Satish K. Kedia:** Writing – review & editing. **Xinhua Yu:** Conceptualization, Data curation, Methodology, Software, Supervision, Validation, Writing – review & editing.

## Appendix Supplementary materials

Image, application 1

## Footnotes

Supplementary material associated with this article can be found, in the online version, at doi:10.1016/j.focus.2023.100175.
