# Supplementary material for: Burden of Chronic and Heavy Opioid Use Among Elderly Community Dwellers in the U.S
Source: AJPM Focus. 2023 Dec 20;3(2):100175. doi: 10.1016/j.focus.2023.100175 (PMC10828592; doi:10.1016/j.focus.2023.100175)
Supplement: Supplementary file 1 [file mmc1.docx]

**Fig 1a-c.** Median total opioid dosage during a continuous treatment episode measured in morphine milligram equivalence (MME) for ever use, chronic, and heavy use by gender from 2006 to 2019.

**Fig 2a-c.** Median opioid duration (in days) for ever use, chronic, and heavy use by gender from 2006 to 2019.

**Fig 3a-c.** Median total opioid dosage during a continuous treatment episode measured in morphine milligram equivalence (MME) ever use, chronic, and heavy use by age from 2006 to 2019.

**Fig 4a-c.** Median opioid duration (in days) for ever use, chronic, and heavy use by age from 2006 to 2019.

**Fig 5a-c.** Median total opioid dosage during a continuous treatment episode measured in morphine milligram equivalence (MME) ever use, chronic, and heavy use by race from 2006 to 2019.

**Fig 6a-c.** Median opioid duration (in days) for ever use, chronic, and heavy use by race from 2006 to 2019.

**Fig 7a-c.** Median total opioid dosage during a continuous treatment episode measured in morphine milligram equivalence (MME) ever use, chronic, and heavy use by income from 2006 to 2019.

**Fig 8a-c.** Median opioid duration (in days) for ever use, chronic, and heavy use by income from 2006 to 2019.

**Fig 9a-c.** Median total opioid dosage during a continuous treatment episode measured in morphine milligram equivalence (MME) ever use, chronic, and heavy use by comorbidities from 2006 to 2019.

**Fig 10a-c.** Median opioid duration (in days) for ever use, chronic, and heavy use by comorbidities from 2006 to 2019.

**Fig 11.** Single Interrupted Time Series Analysis (SITSA) of Opioid Use Rates between 2006 to 2019.

**Fig 1a.** Median total opioid dosage during a continuous treatment episode measured in morphine milligram equivalence (MME) ever use by gender from 2006 to 2019.


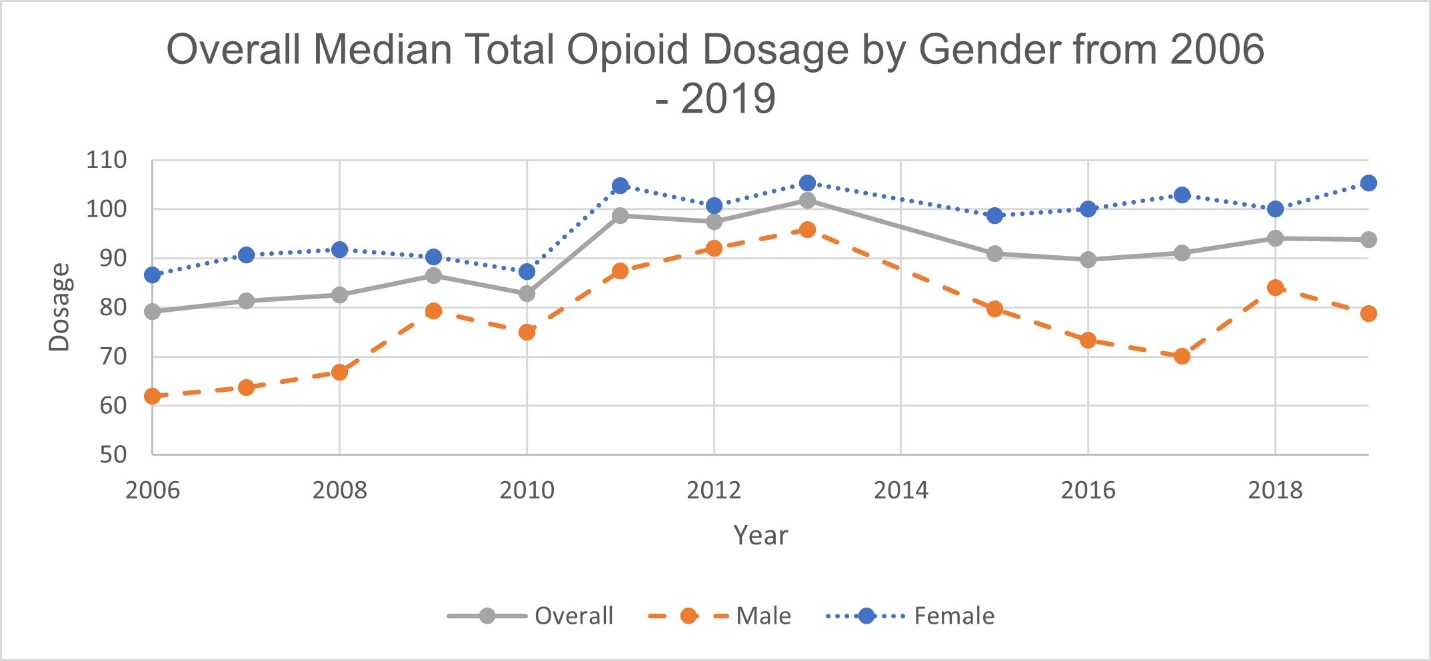


**Fig 1b.** Median total opioid dosage during a continuous treatment episode measured in morphine milligram equivalence (MME) chronic use by gender from 2006 to 2019.


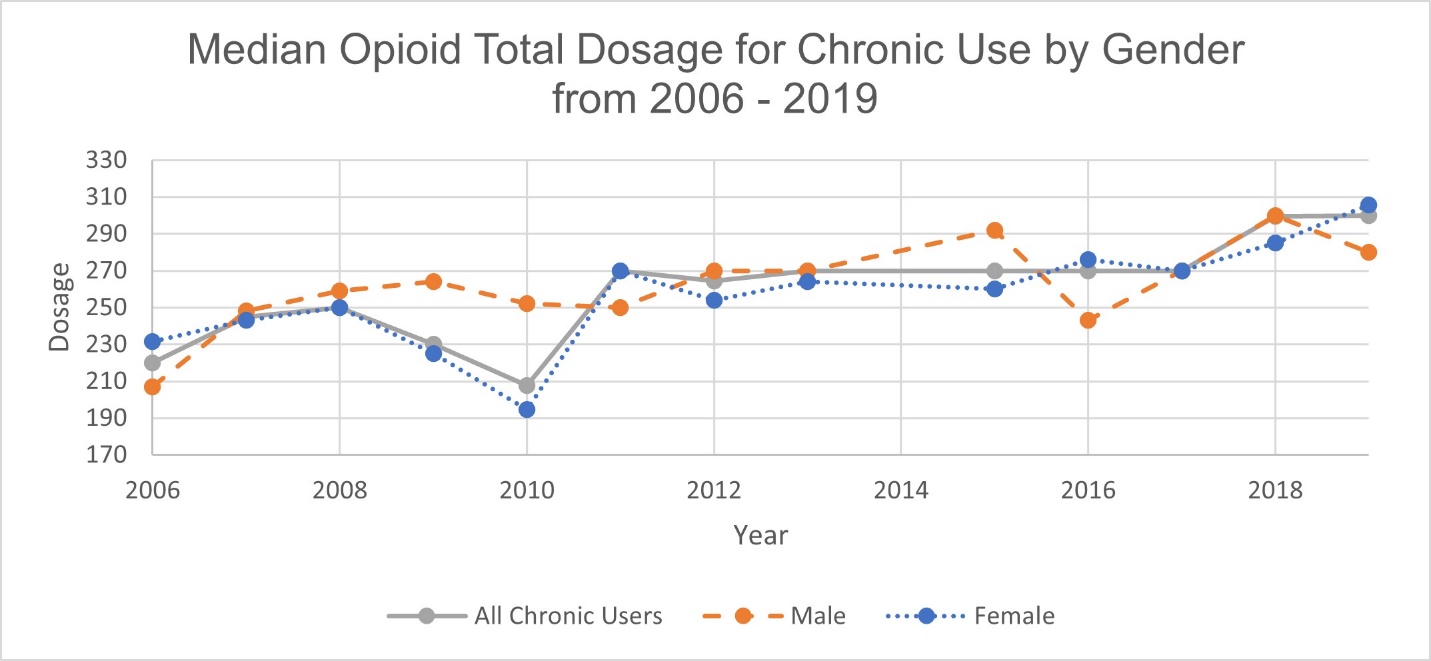


**Fig 1c.** Median total opioid dosage during a continuous treatment episode measured in morphine milligram equivalence (MME) heavy use by gender from 2006 to 2019.


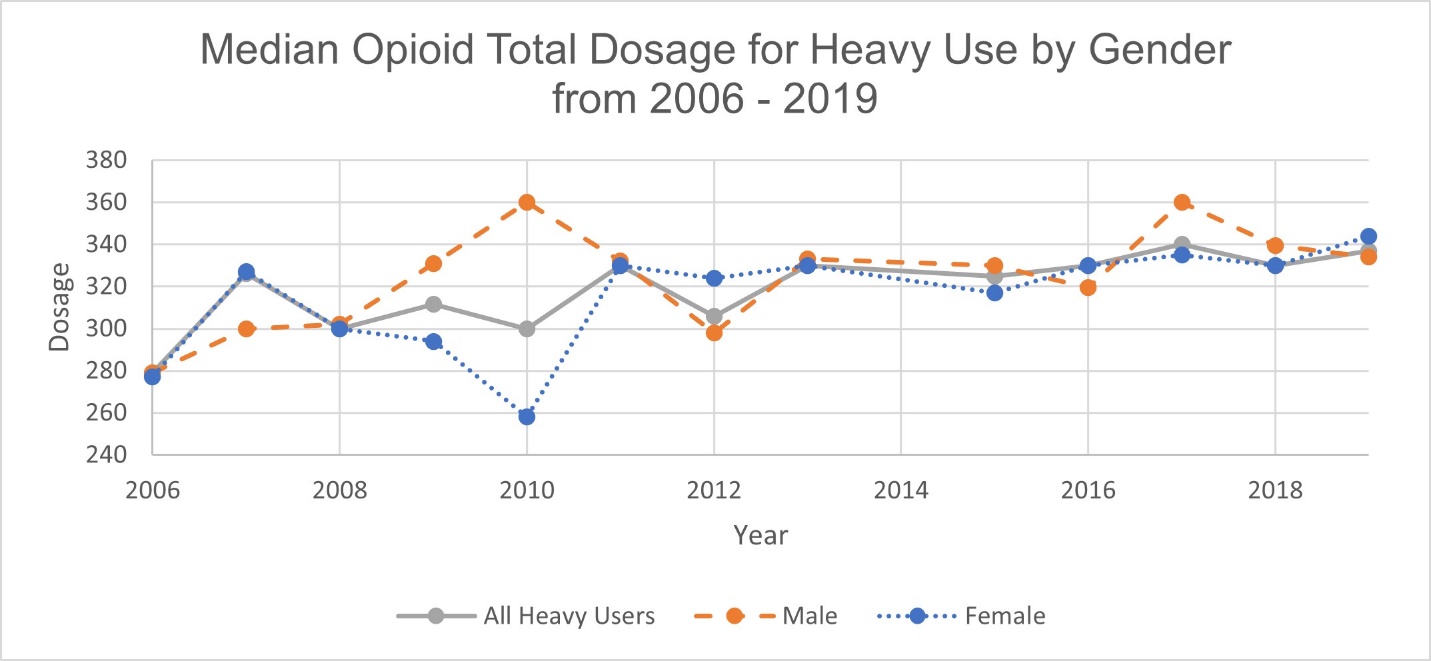


**Fig 2a.** Median opioid duration (in days) for ever use by gender from 2006 to 2019.


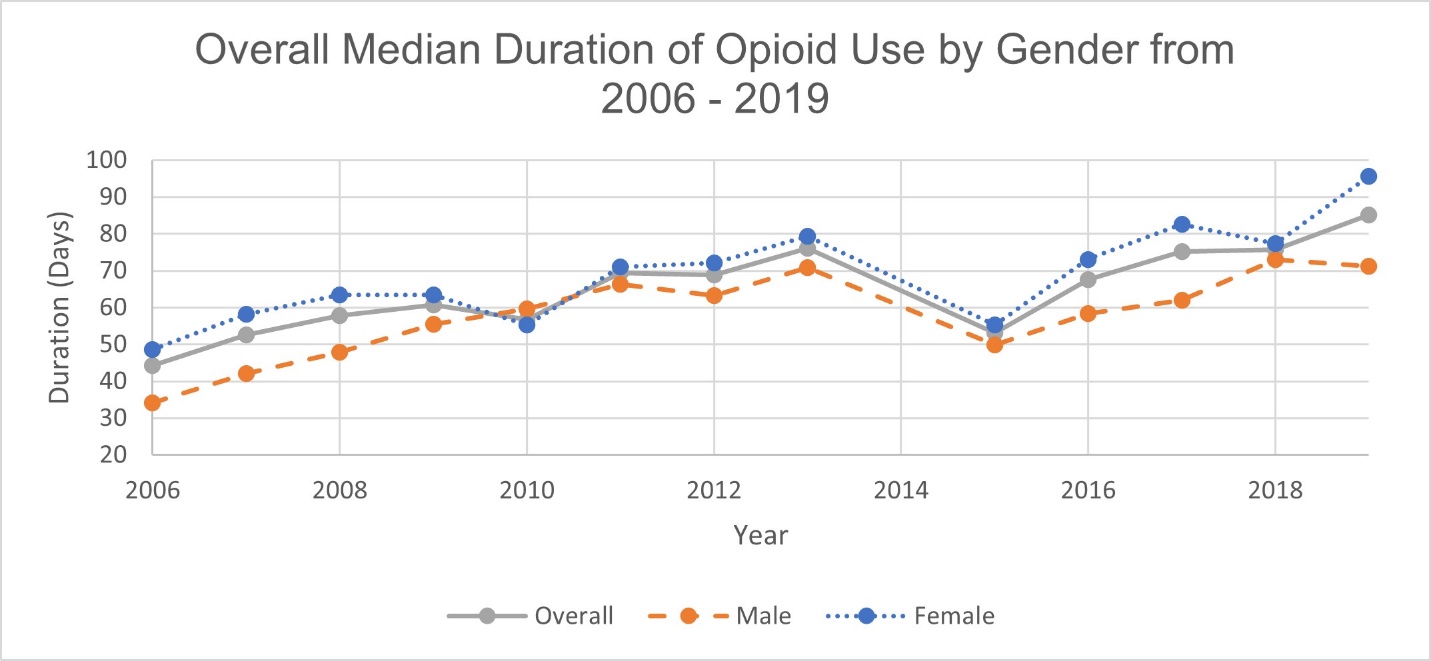


**Fig 2b.** Median opioid duration (in days) for chronic by gender from 2006 to 2019.


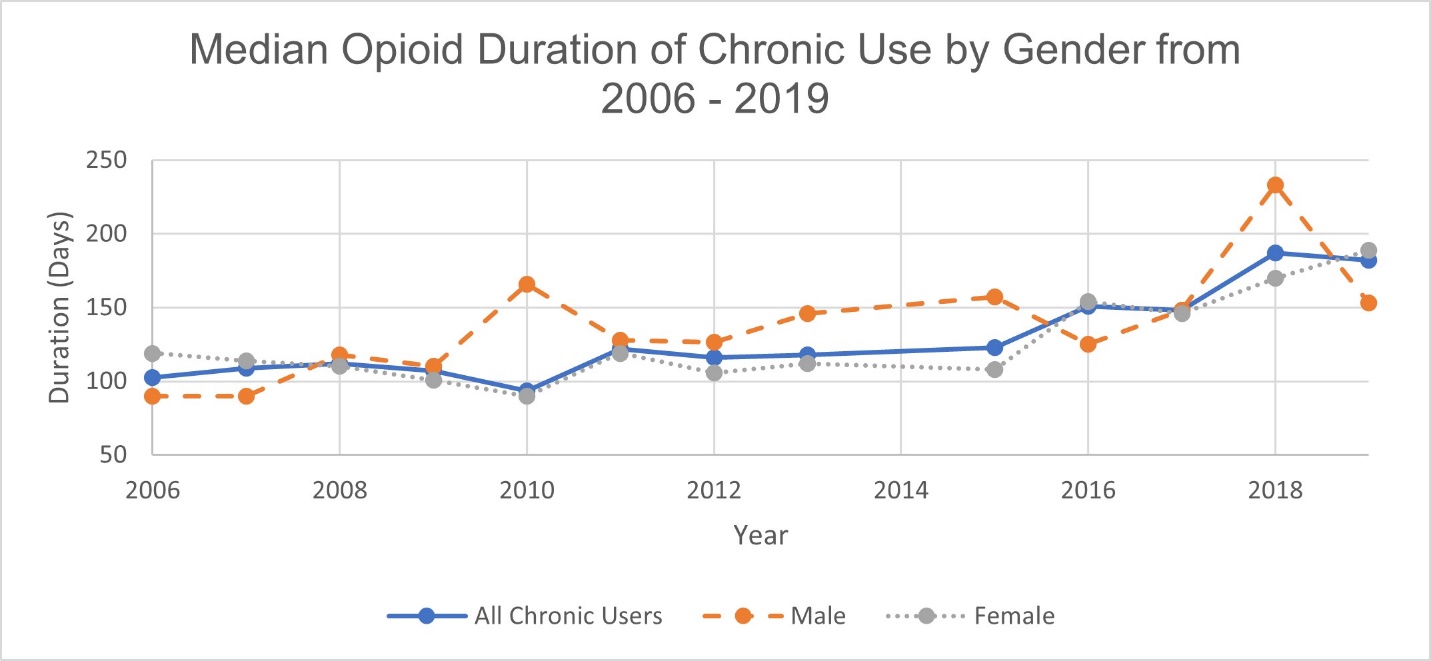


**Fig 2c.** Median opioid duration (in days) for heavy use by gender from 2006 to 2019.


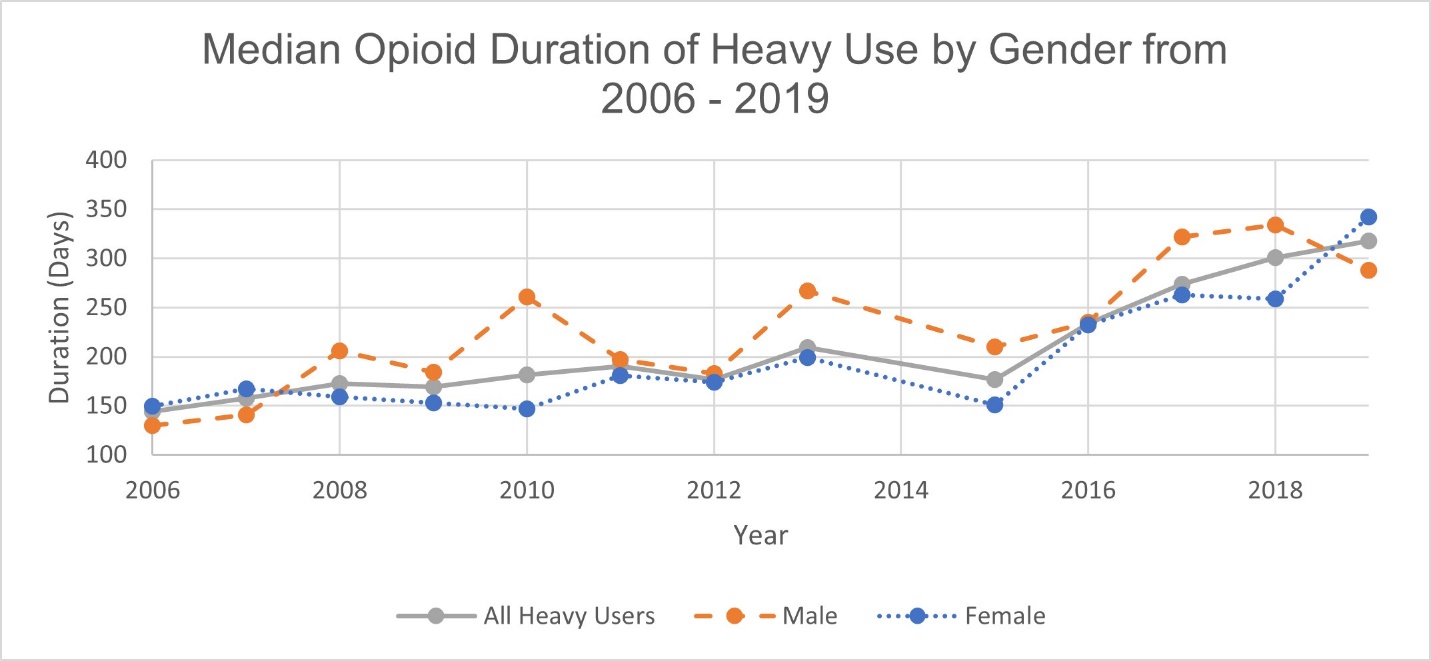


**Fig 3a.** Median total opioid dosage during a continuous treatment episode measured in morphine milligram equivalence (MME) ever use by age from 2006 to 2019.


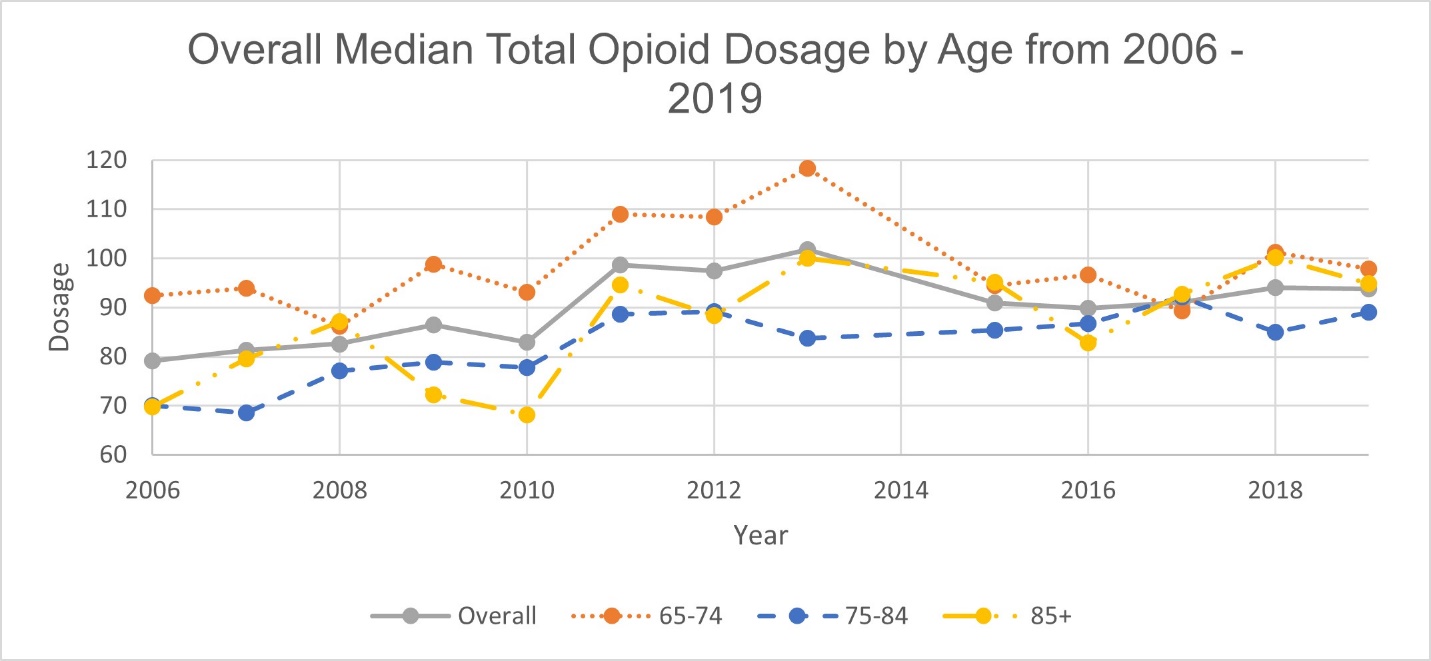


**Fig 3b.** Median total opioid dosage during a continuous treatment episode measured in morphine milligram equivalence (MME) chronic use by age from 2006 to 2019.


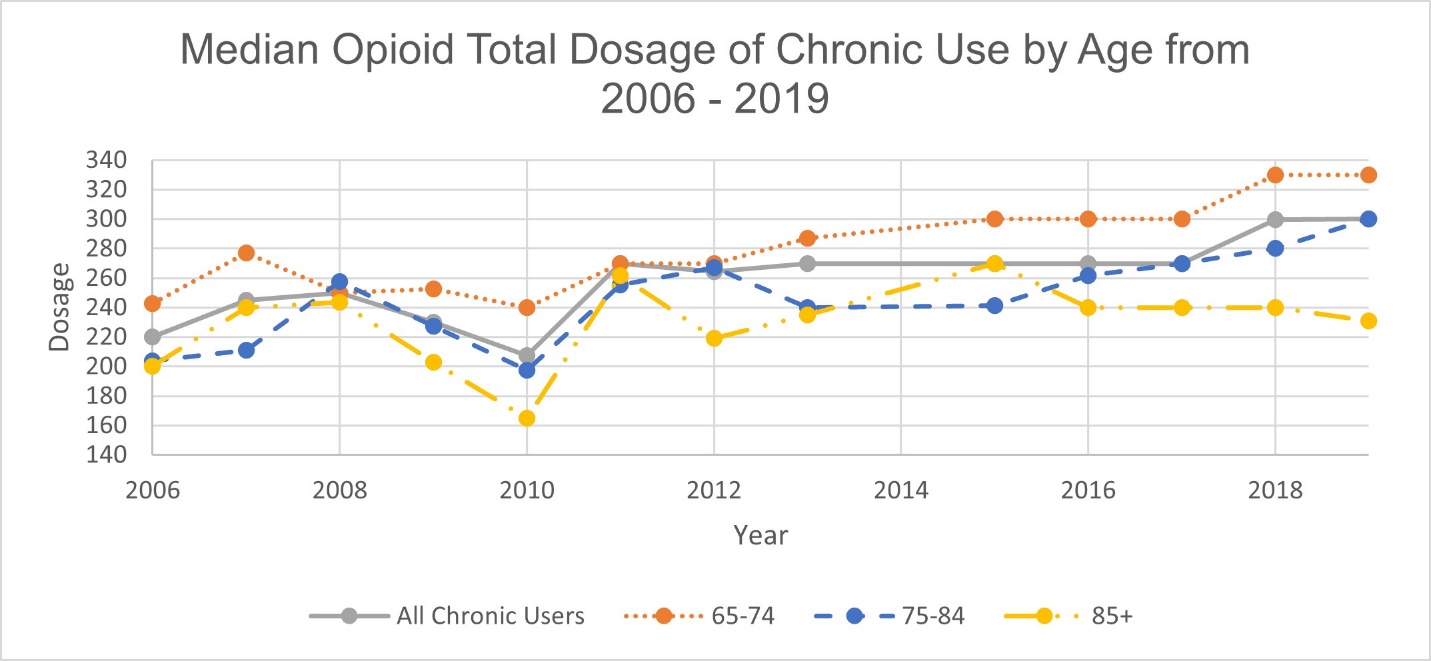


**Fig 3c.** Median total opioid dosage during a continuous treatment episode measured in morphine milligram equivalence (MME) heavy use by age from 2006 to 2019.


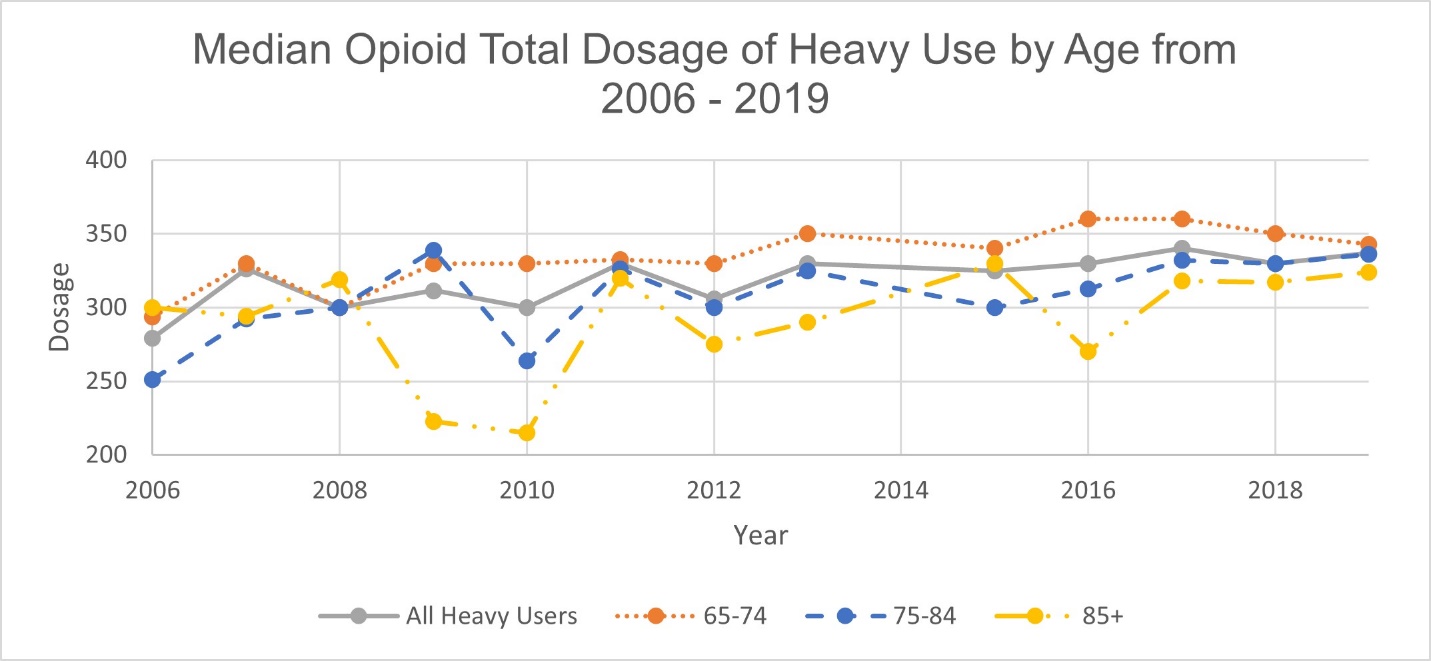


**Fig 4a.** Median opioid duration (in days) for ever use by age from 2006 to 2019.


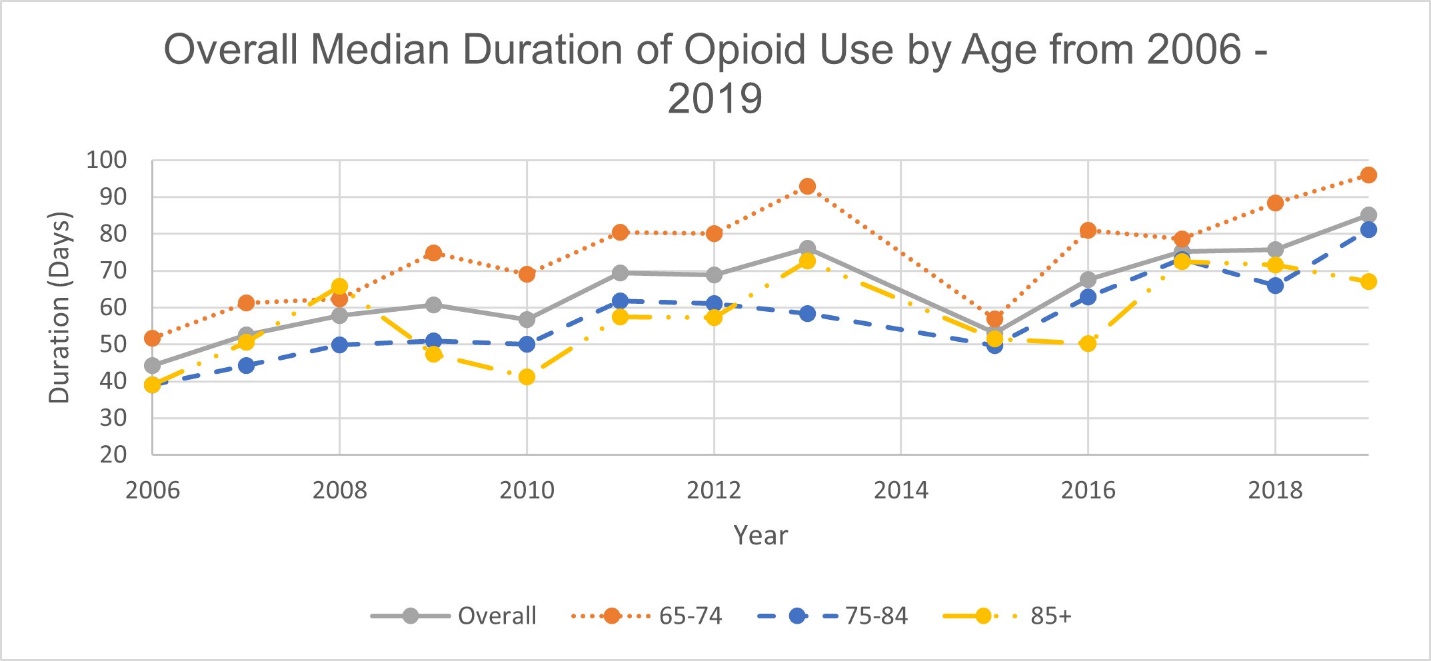


**Fig 4b.** Median opioid duration (in days) for chronic use by age from 2006 to 2019.


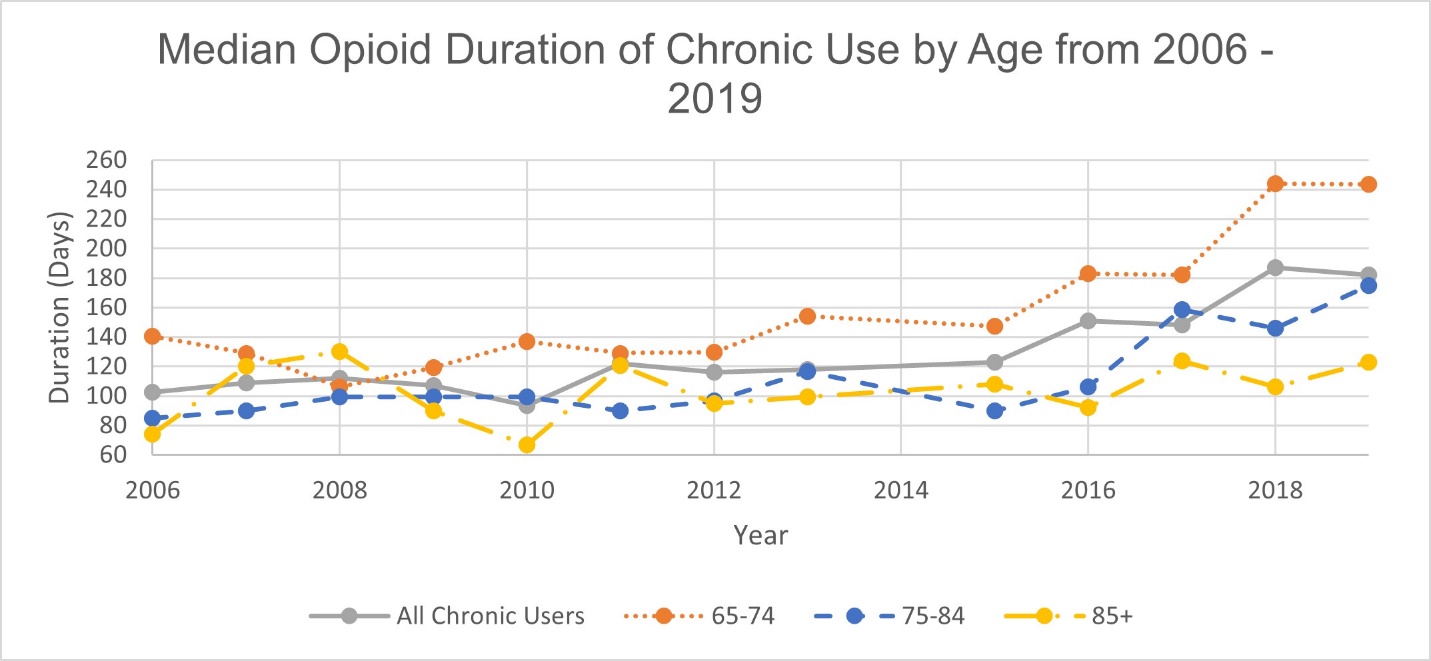


**Fig 4c.** Median opioid duration (in days) for heavy use by age from 2006 to 2019.


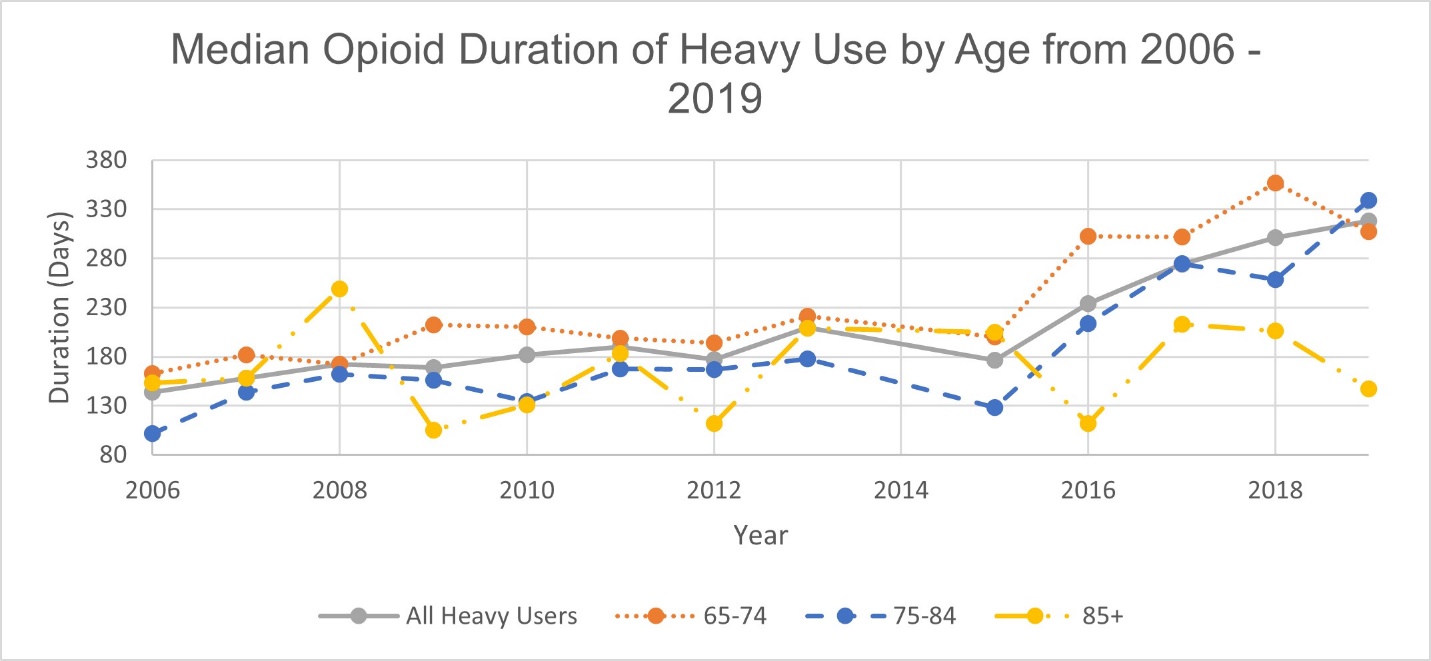


**Fig 5a.** Median total opioid dosage during a continuous treatment episode measured in morphine milligram equivalence (MME) ever use by race from 2006 to 2019.


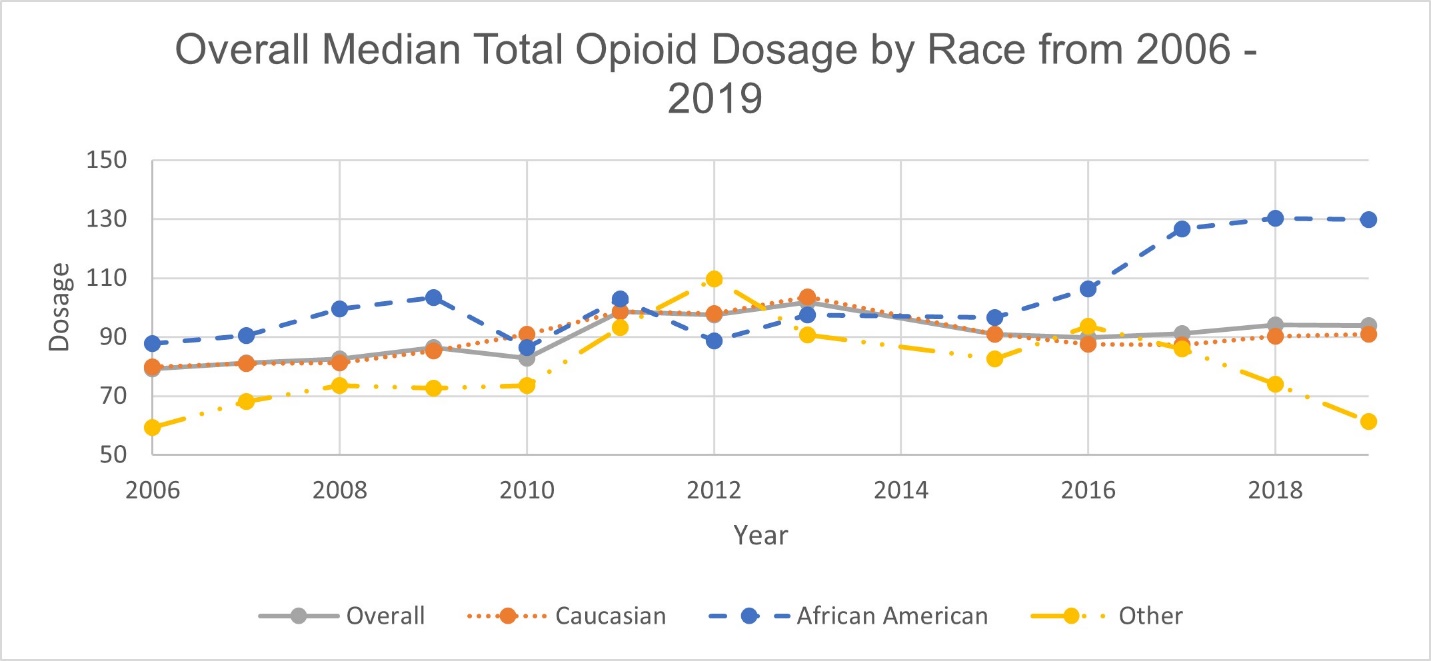


**Fig 5b.** Median total opioid dosage during a continuous treatment episode measured in morphine milligram equivalence (MME) chronic use by race from 2006 to 2019.


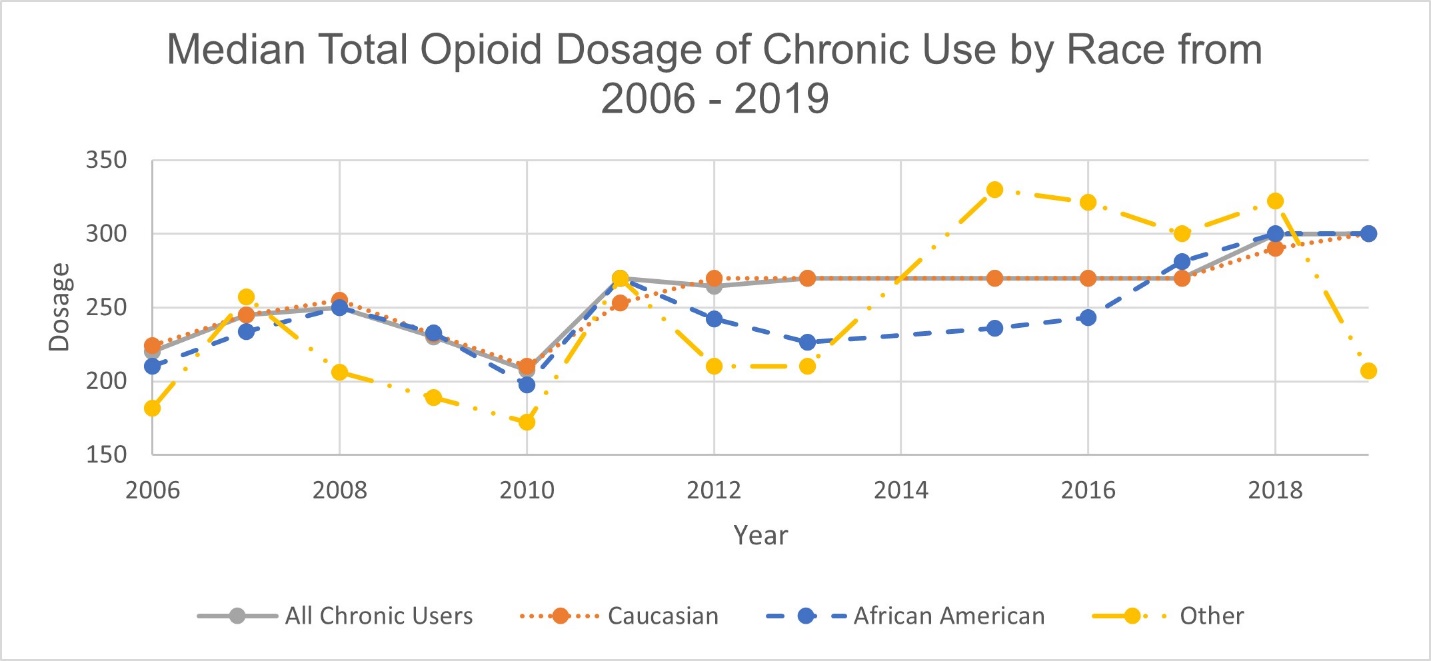


**Fig 5c.** Median total opioid dosage during a continuous treatment episode measured in morphine milligram equivalence (MME) heavy use by race from 2006 to 2019.


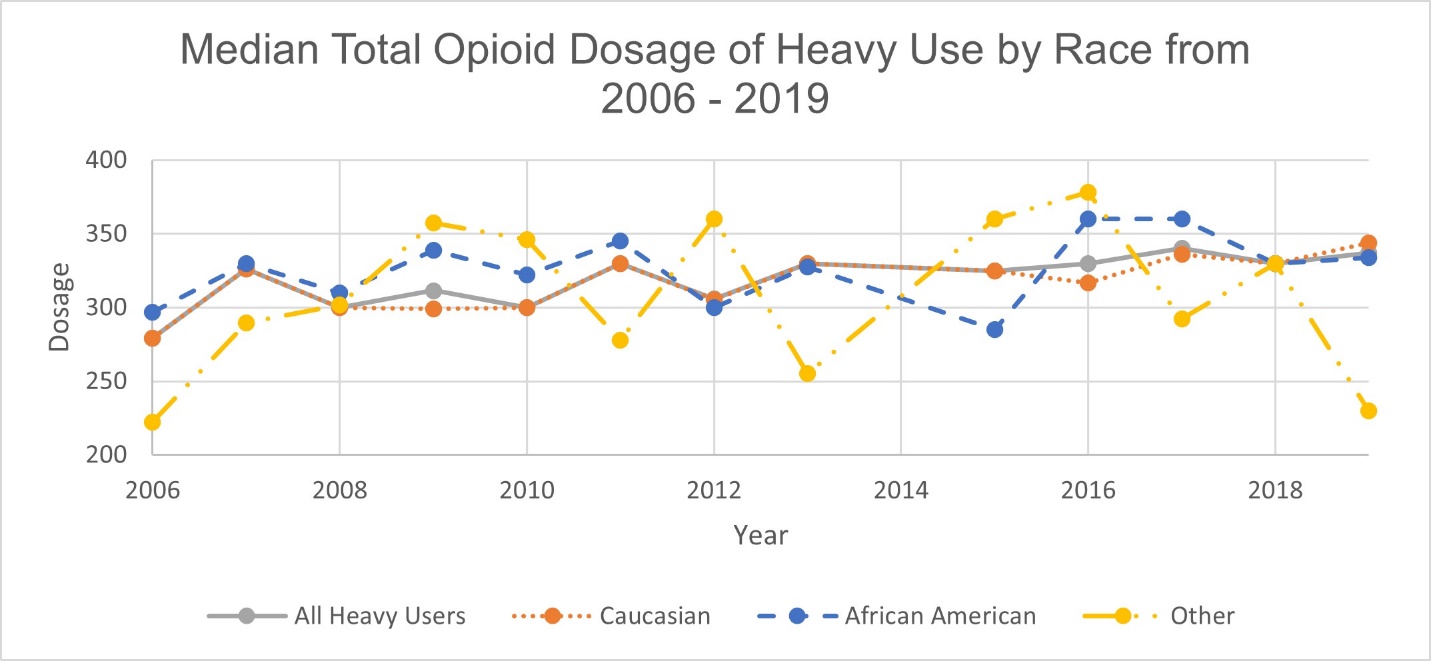


**Fig 6a.** Median opioid duration (in days) for ever use by race from 2006 to 2019.


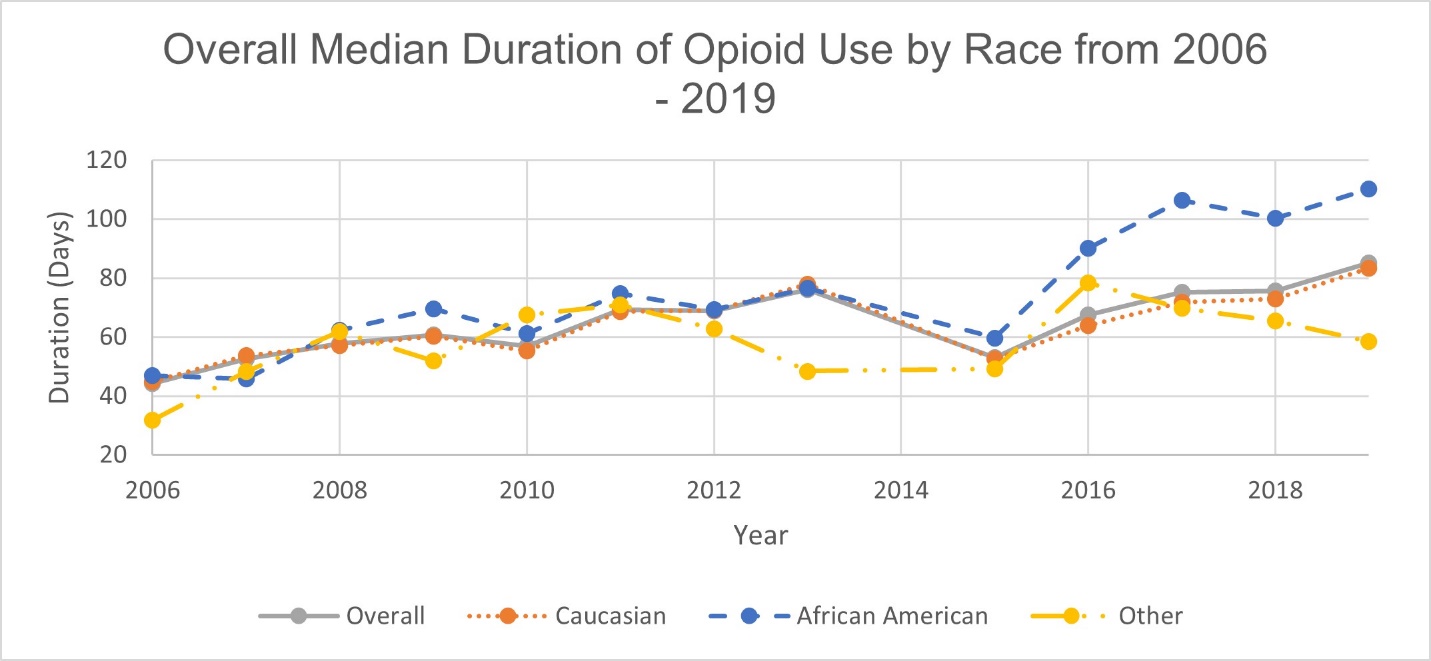


**Fig 6b.** Median opioid duration (in days) for chronic use by race from 2006 to 2019.


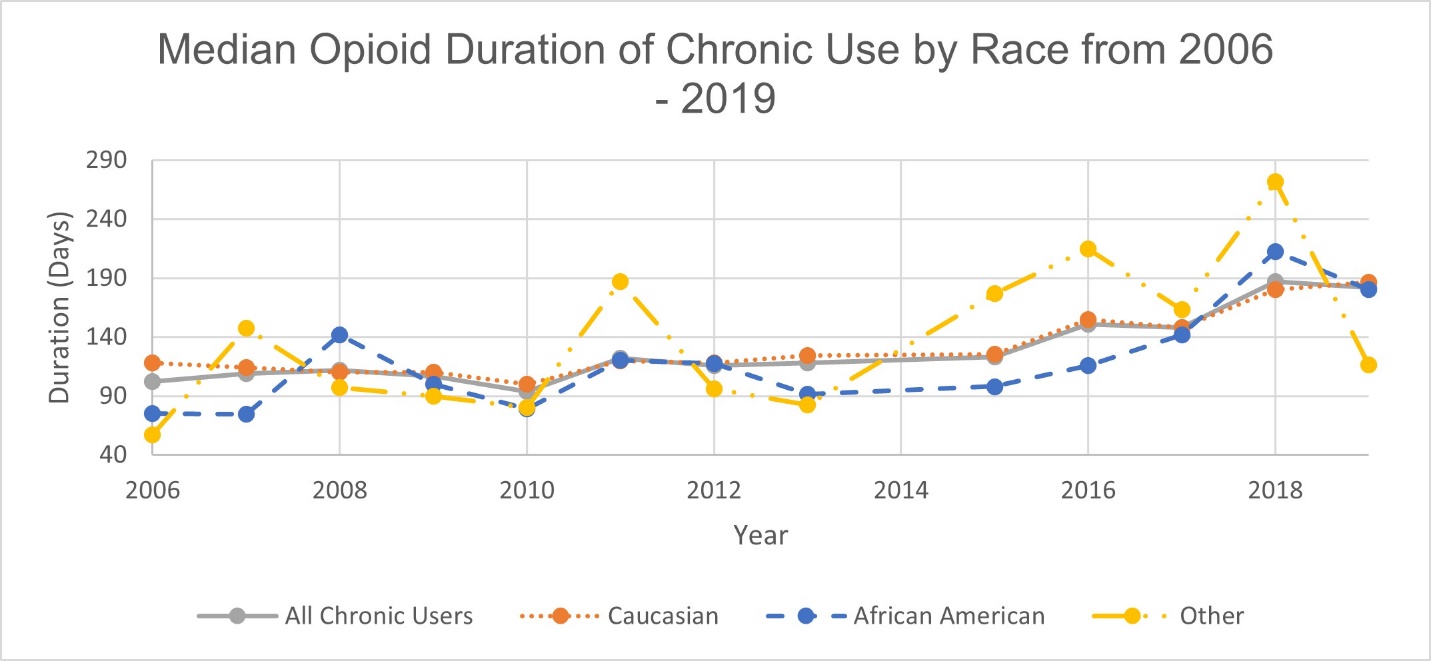


**Fig 6c.** Median opioid duration (in days) for heavy use by race from 2006 to 2019.


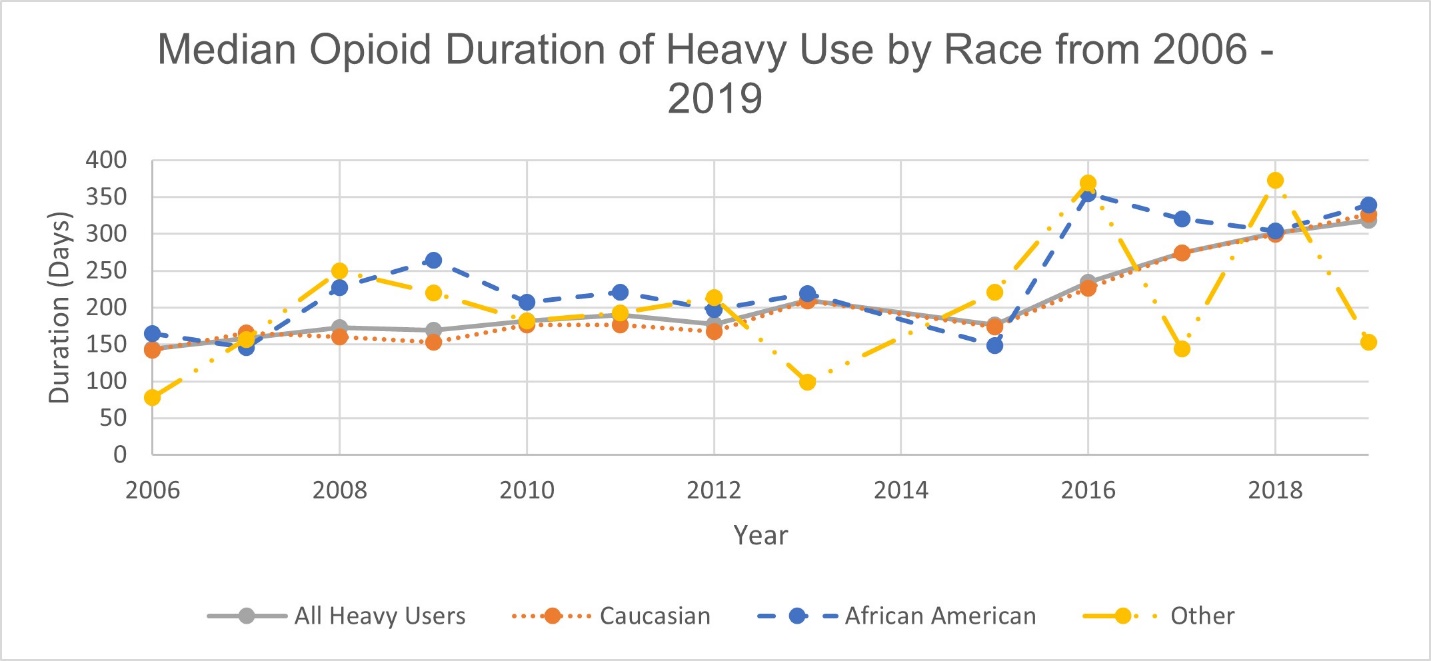


**Fig 7a.** Median total opioid dosage during a continuous treatment episode measured in morphine milligram equivalence (MME) ever use by income from 2006 to 2019.


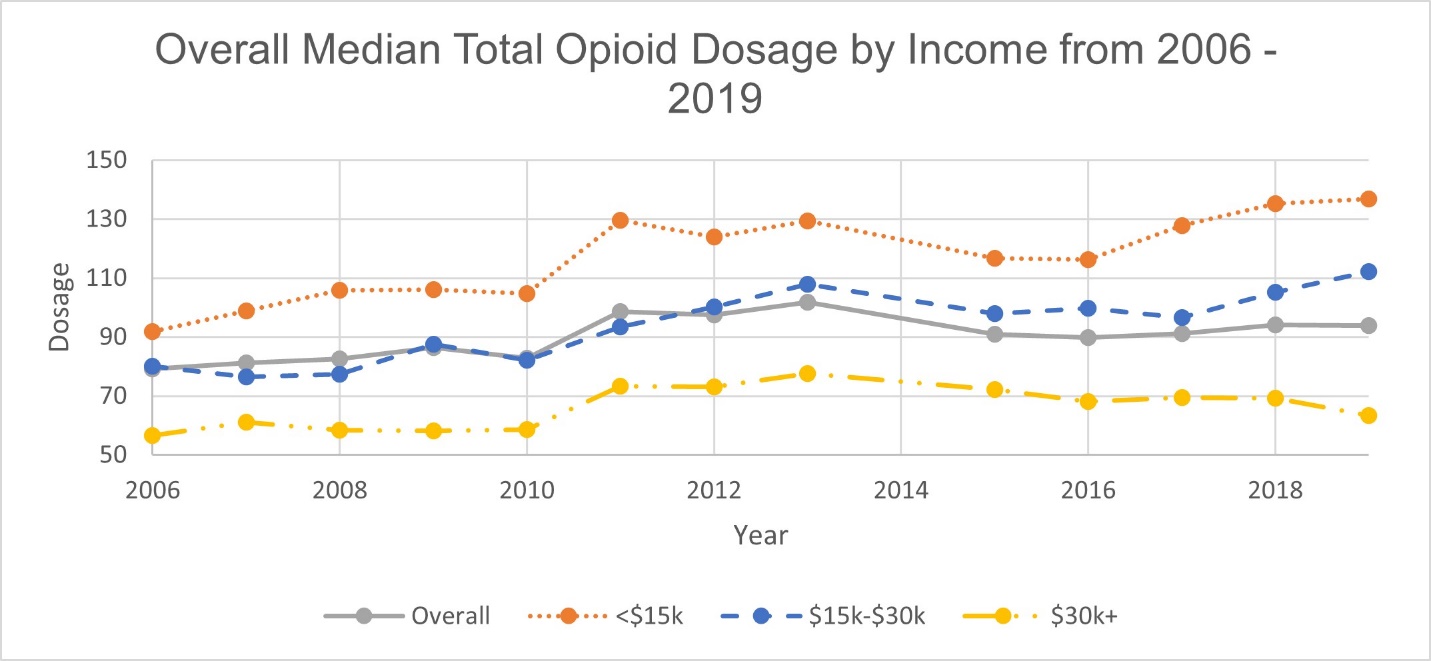


**Fig 7b.** Median total opioid dosage during a continuous treatment episode measured in morphine milligram equivalence (MME) chronic use by income from 2006 to 2019.


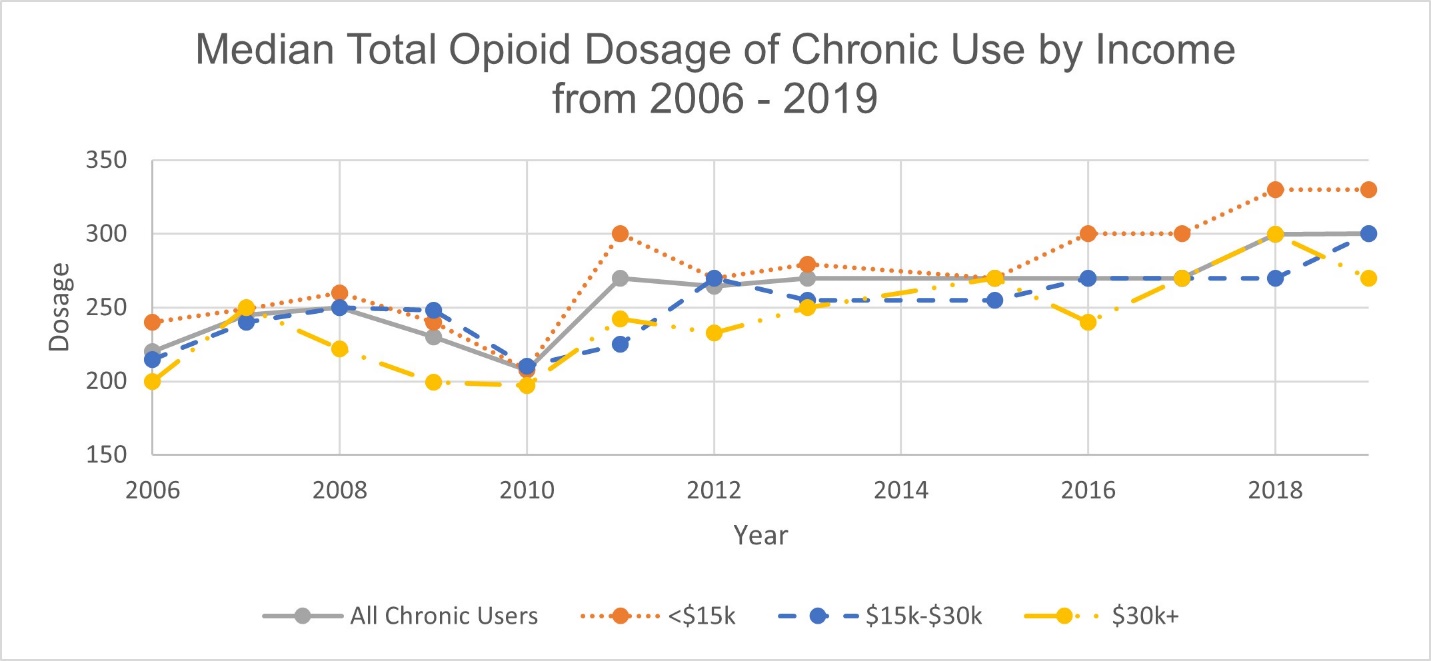


**Fig 7c.** Median total opioid dosage during a continuous treatment episode measured in morphine milligram equivalence (MME) heavy use by income from 2006 to 2019.


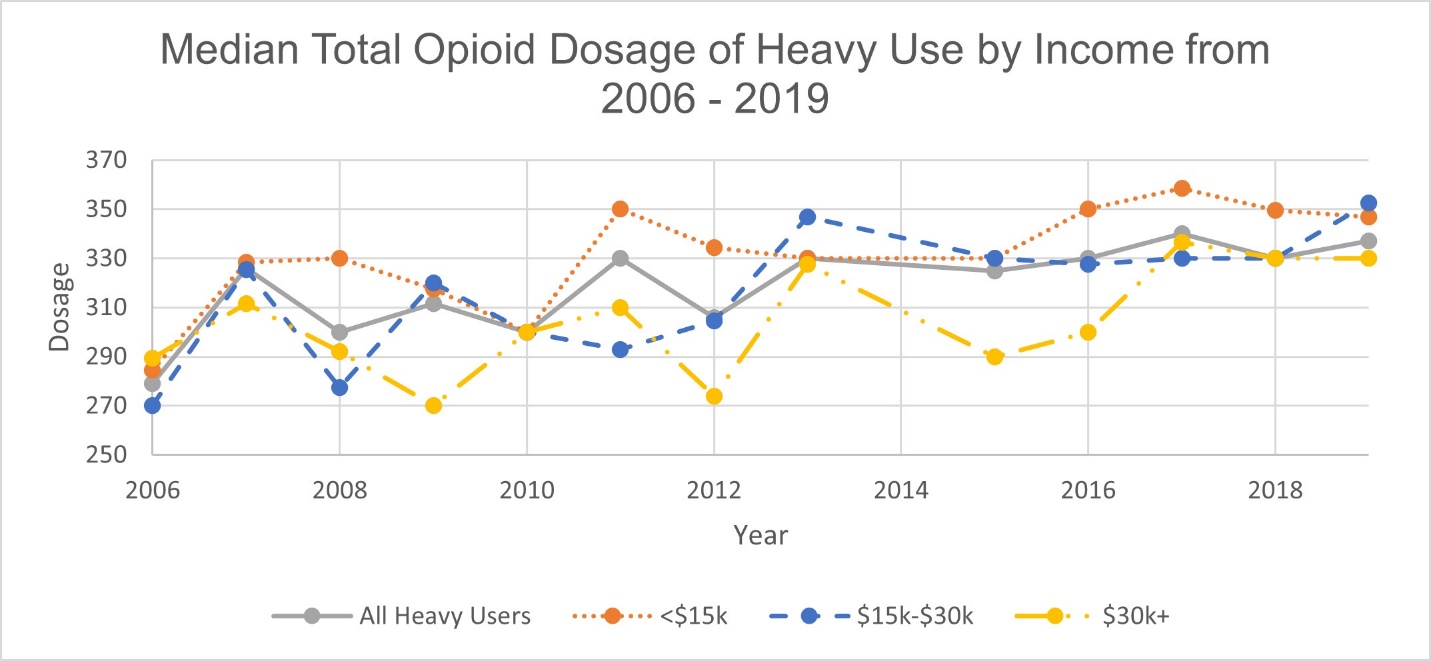


**Fig 8a.** Median opioid duration (in days) for ever use by income from 2006 to 2019.


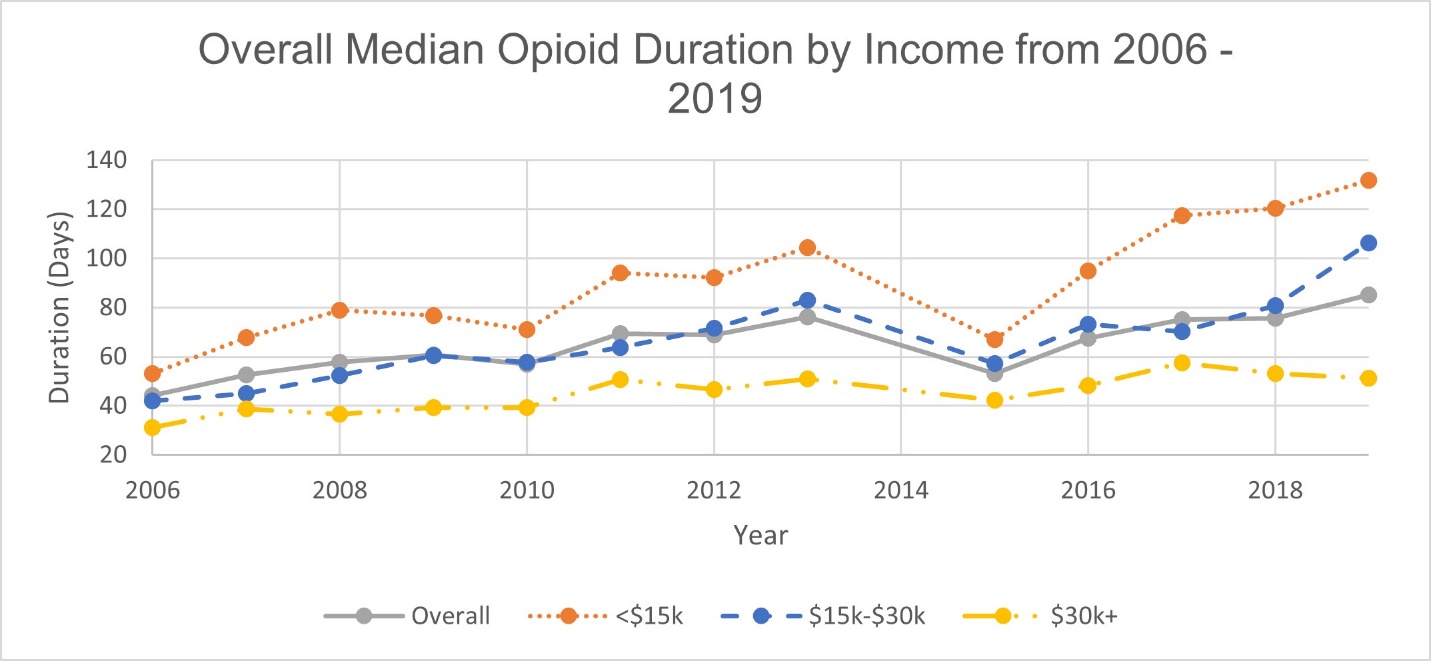


**Fig 8b.** Median opioid duration (in days) for chronic use by income from 2006 to 2019.


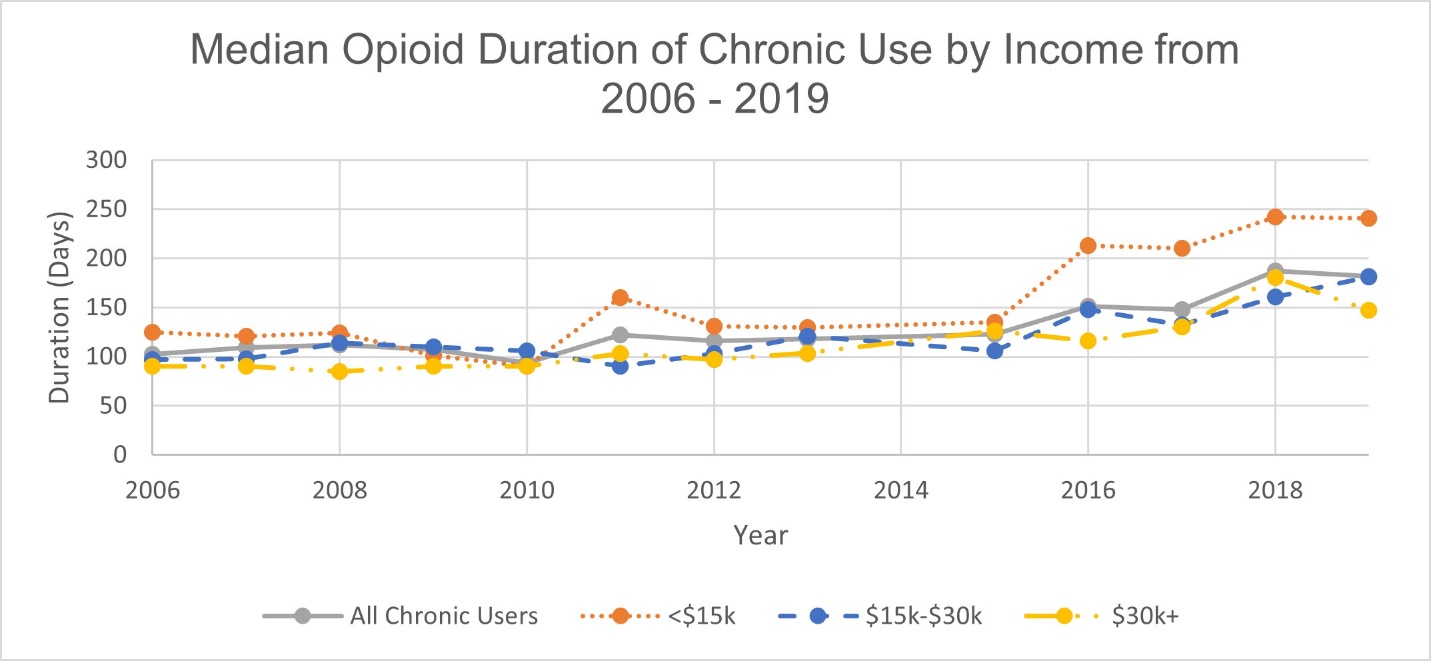


**Fig 8c.** Median opioid duration (in days) for heavy use by income from 2006 to 2019.


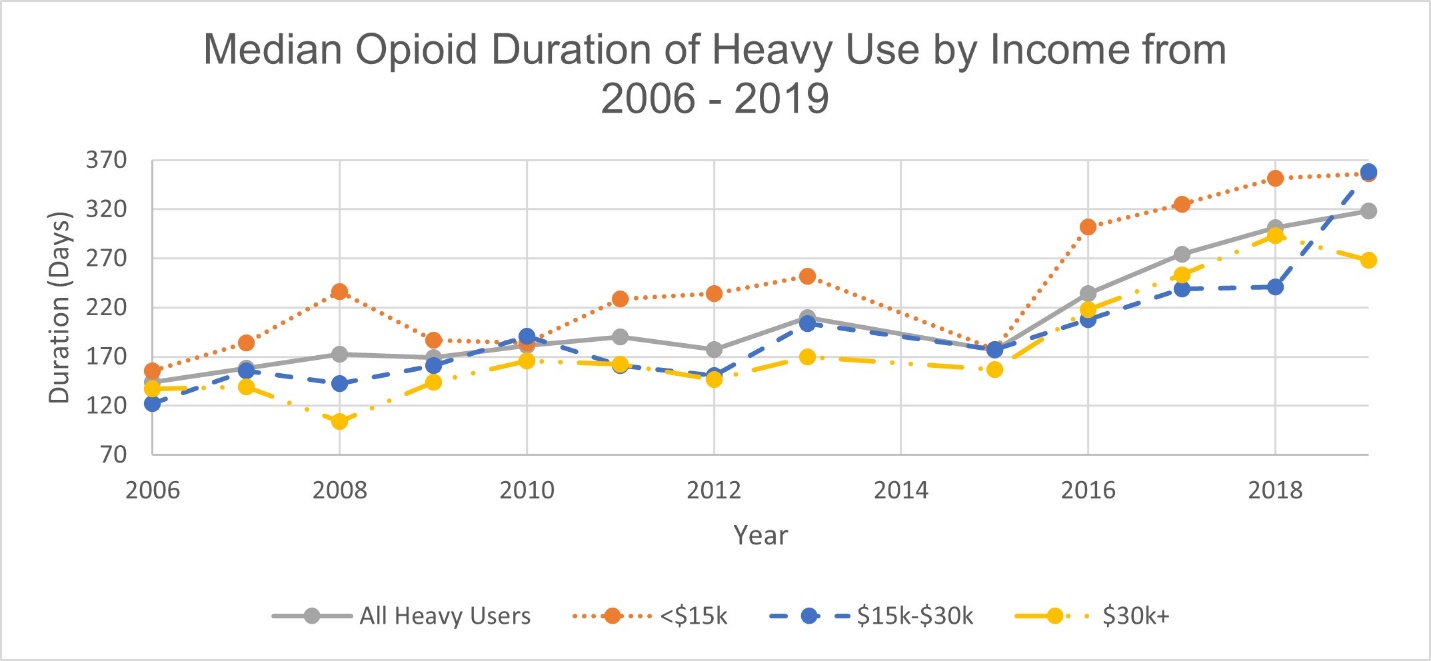


**Fig 9a.** Median total opioid dosage during a continuous treatment episode measured in morphine milligram equivalence (MME) ever use by comorbidities from 2006 to 2019.


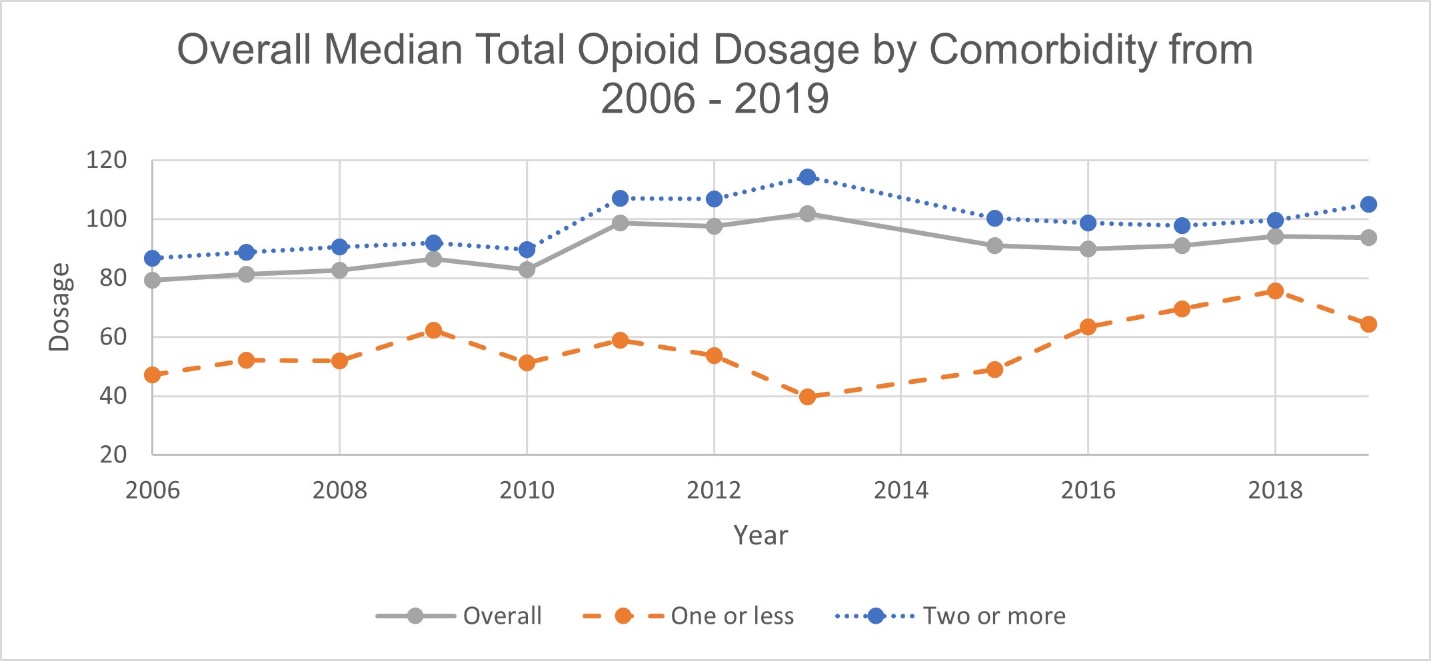


**Fig 9b.** Median total opioid dosage during a continuous treatment episode measured in morphine milligram equivalence (MME) chronic use by comorbidities from 2006 to 2019.


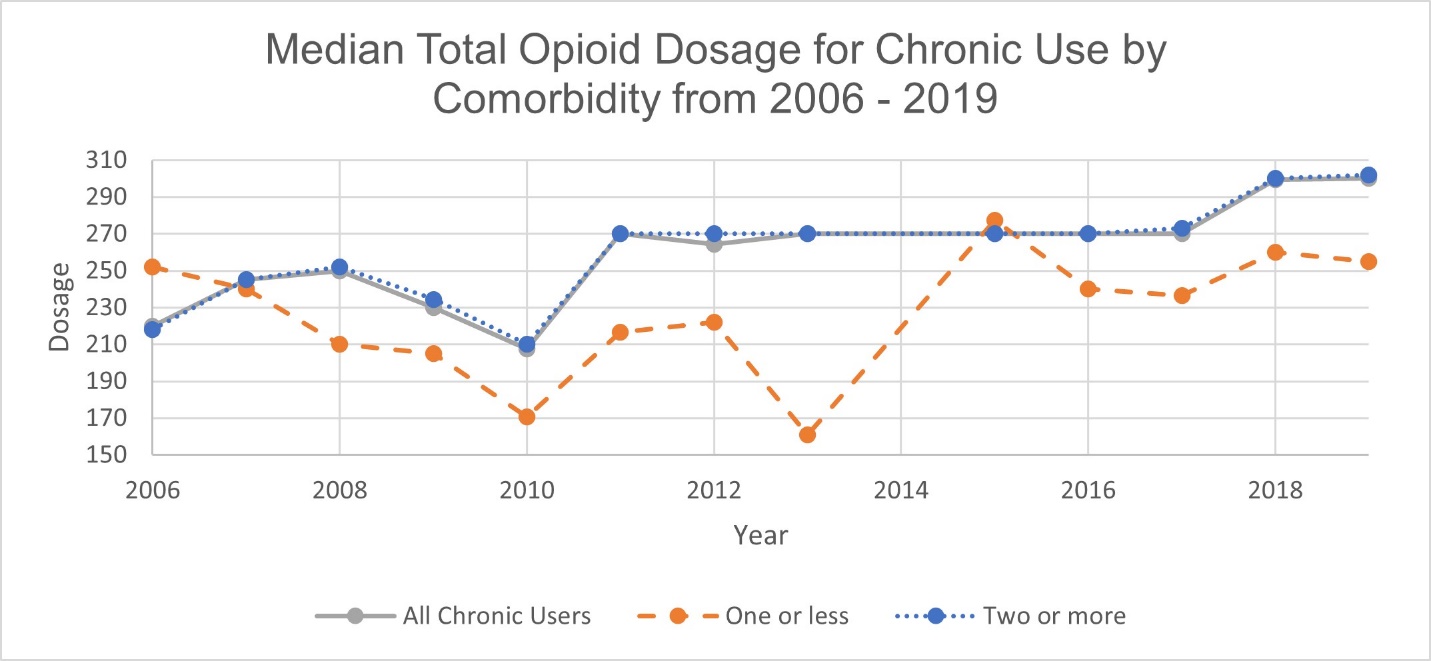


**Fig 9c.** Median total opioid dosage during a continuous treatment episode measured in morphine milligram equivalence (MME) heavy use by comorbidities from 2006 to 2019.


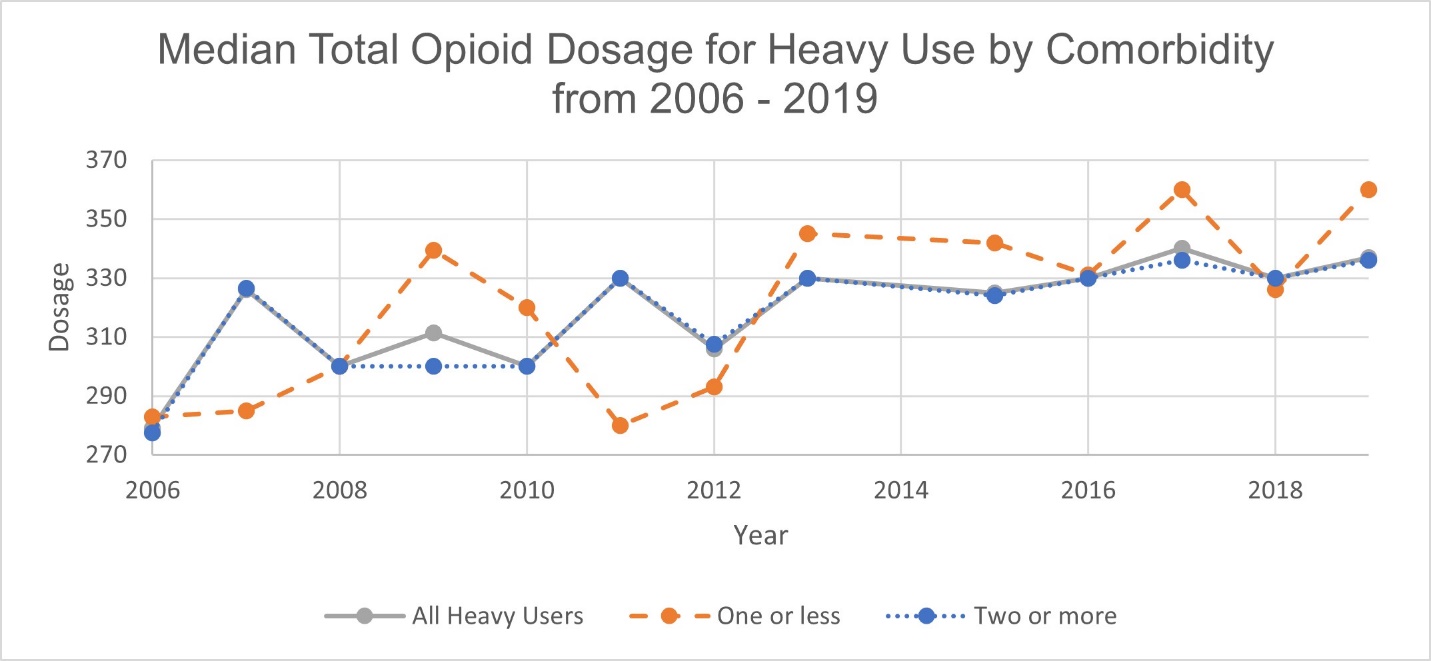


**Fig 10a.** Median opioid duration (in days) for ever use by comorbidities from 2006 to 2019.


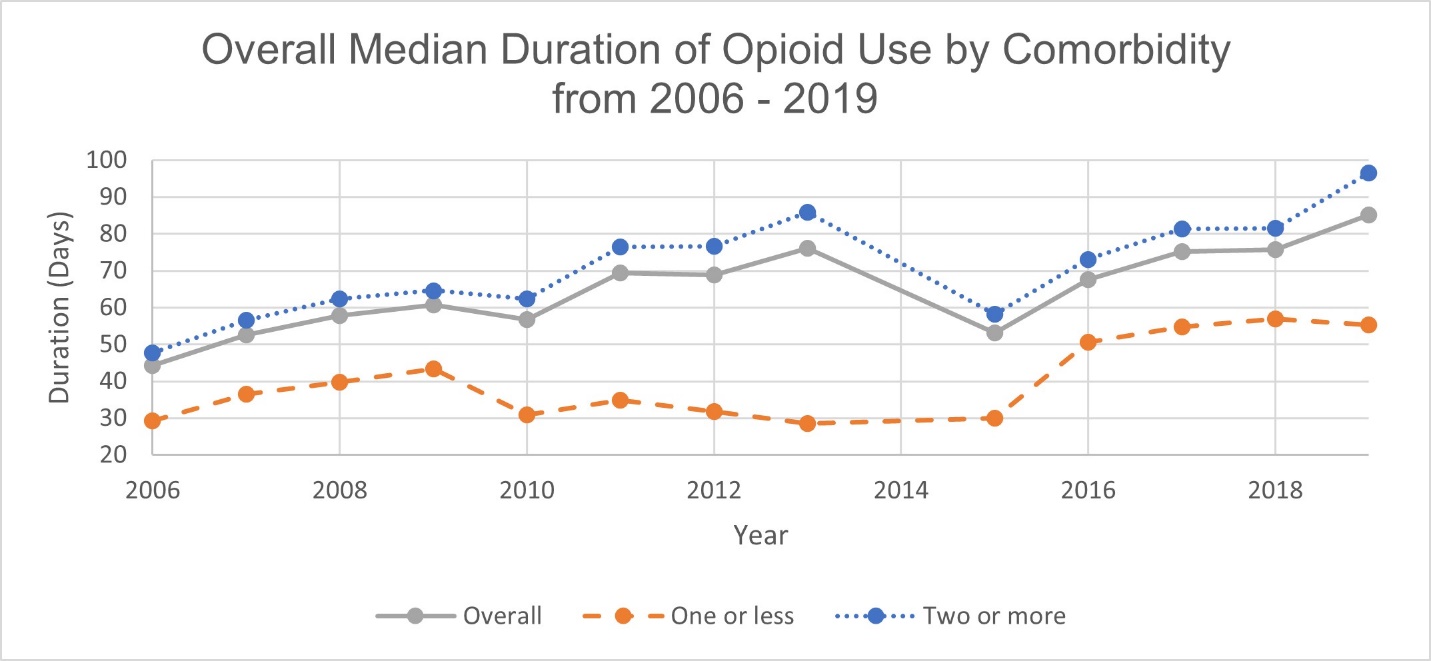


**Fig 10b.** Median opioid duration (in days) for chronic use by comorbidities from 2006 to 2019.


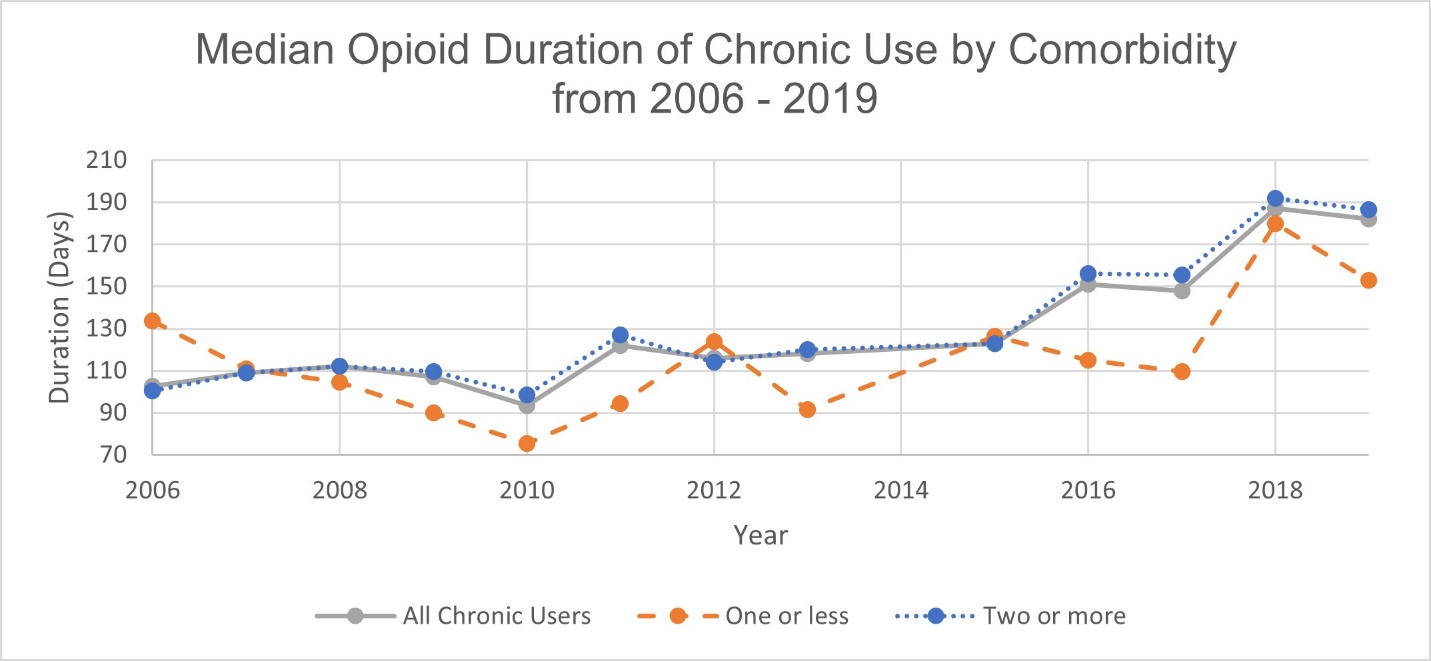


**Fig 10c.** Median opioid duration (in days) for heavy use by comorbidities from 2006 to 2019.


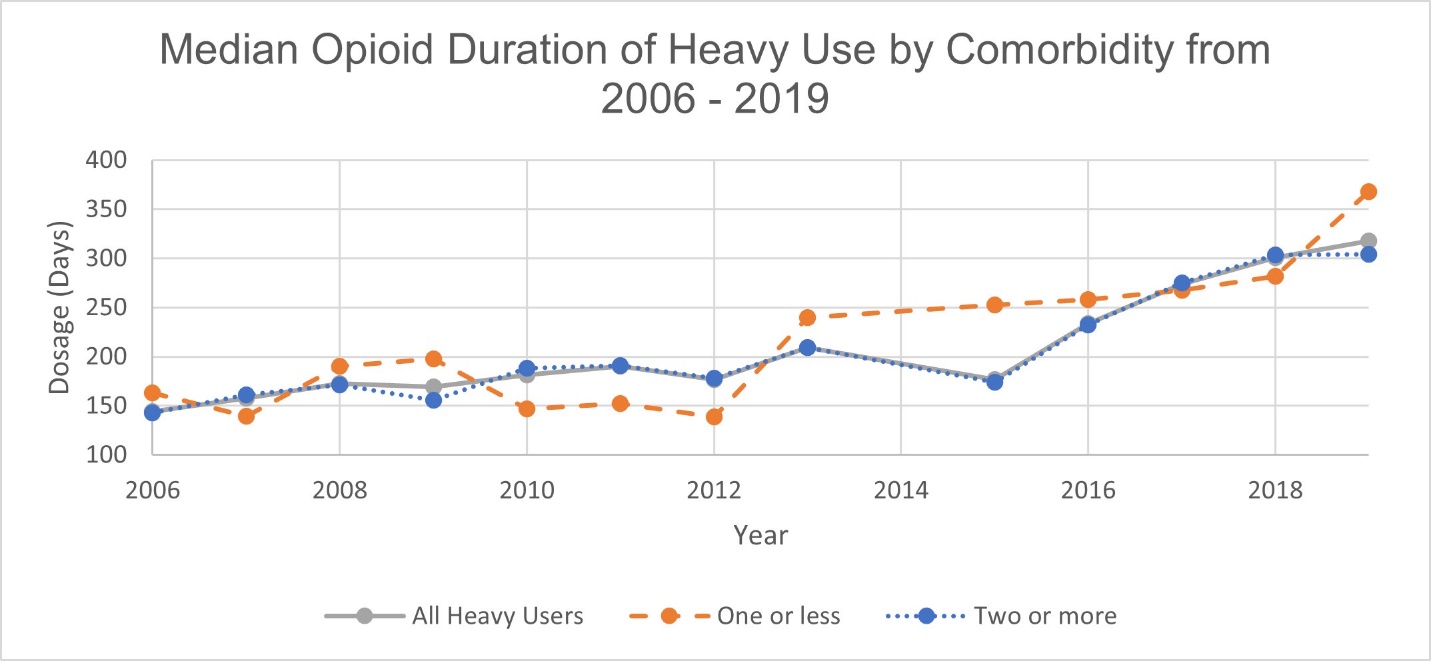


**Fig 11.** Single Interrupted Time Series Analysis (SITSA) of Opioid Use Rates between 2006 to 2019.

**
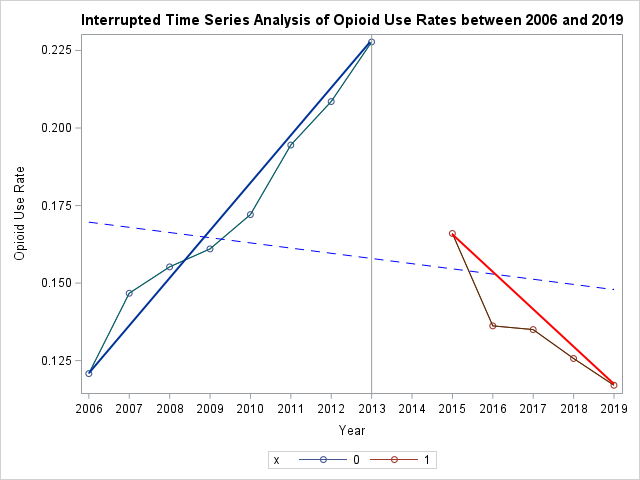
**
